# Electrochemical polymerization of pyrene derivatives on functionalized carbon nanotubes for pseudocapacitive electrodes

**DOI:** 10.1038/ncomms8040

**Published:** 2015-05-06

**Authors:** John C. Bachman, Reza Kavian, Daniel J. Graham, Dong Young Kim, Suguru Noda, Daniel G. Nocera, Yang Shao-Horn, Seung Woo Lee

**Affiliations:** 1 Department of Mechanical Engineering, Massachusetts Institute of Technology, Cambridge, MA 02139, USA; 2 George W. Woodruff School of Mechanical Engineering, Georgia Institute of Technology, Atlanta, GA 30332, USA; 3 Department of Chemistry and Chemical Biology, Harvard University, Cambridge, MA 02138, USA; 4 Department of Applied Chemistry, Waseda University, 3-4-1 Okubo, Shinjuku-ku, Tokyo 169-8555, Japan; 5 Department of Materials Science and Engineering, Massachusetts Institute of Technology, Cambridge, MA 02139, USA

## Abstract

Electrochemical energy-storage devices have the potential to be clean and efficient, but their current cost and performance limit their use in numerous transportation and stationary applications. Many organic molecules are abundant, economical and electrochemically active; if selected correctly and rationally designed, these organic molecules offer a promising route to expand the applications of these energy-storage devices. In this study, polycyclic aromatic hydrocarbons are introduced within a functionalized few-walled carbon nanotube matrix to develop high-energy, high-power positive electrodes for pseudocapacitor applications. The reduction potential and capacity of various polycyclic aromatic hydrocarbons are correlated with their interaction with the functionalized few-walled carbon nanotube matrix, chemical configuration and electronic structure. These findings provide rational design criteria for nanostructured organic electrodes. When combined with lithium negative electrodes, these nanostructured organic electrodes exhibit energy densities of ∼350 Wh kg^−1^_electrode_ at power densities of ∼10 kW kg^−1^_electrode_ for over 10,000 cycles.

## Abstract (web summary)

Electrochemically active organic molecules are an important class of electrode materials for energy storage. Here, the authors report organic electrodes made of polycyclic aromatic hydrocarbons and functionalized few-walled carbon nanotubes, which show promising electrochemical performance.

## Graphical abstract

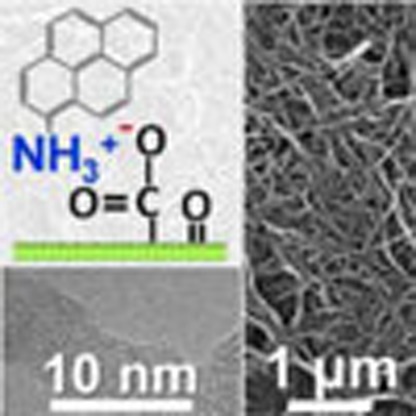

Inexpensive, scalable and reliable electrical energy storage is essential for the wide-spread introduction of large-scale renewable energy generation and the electrification of vehicle fleets. Electrochemical energy-storage (EES) systems are particularly attractive for renewable energy storage because of their short charge/discharge time, high energy-storage efficiency, long cycle life and ease of integration into renewable energy sources^1^,^2^. Lithium rechargeable batteries exhibit the highest gravimetric energy density among EES systems, based on their unit mass or volume, by utilizing Faradaic reactions in the bulk of the active materials. These materials include transition metal oxides (LiMO_2_) or metal phosphates (LiMPO_4_) for the positive electrodes and graphite or Li-metal alloys for negative electrodes^1^,^3^,^4^,^5^. However, large-scale installations of batteries for renewable energy storage and electric vehicles require minimal use of rare and expensive inorganic materials, driving the development of more sustainable organic electrodes to increase energy-storage capacity per unit cost^1^,^6^,^7^,^8^,^9^. Electrochemical capacitors (ECs) represent high-power EES systems by utilizing surface ion adsorption onto high surface area carbon electrodes (double-layer capacitance) or surface redox reactions on metal oxides (pseudocapacitance)^10^,^11^,^12^,^13^,^14^,^15^. ECs are also well suited for renewable energy-storage applications owing to their long cycle life and short charge/discharge time, which allows them to provide power stability under fluctuating wind and solar power generation. However, ECs typically suffer from low energy densities due to the surface-limited energy-storage mechanisms^11^. Considerable research effort has been focused on developing electrochemically active polymer materials^6^,^9^,^16^,^17^,^18^,^19^,^20^,^21^,^22^,^23^. Most of the common conducting polymers, such as polyaniline and polypyrrole, exchange anions with the polymer backbone, sometimes described as a p-doping process^6^,^9^,^16^,^17^,^18^,^19^. These anion insertion reactions can have high reduction potentials over 3 V versus Li/Li^+^, but often suffer from poor cycle stability^6^,^16^,^17^. Recent research efforts have been focused on organic carbonyl compounds utilizing the charge-transfer interaction between the carbonyl oxygen moiety and lithium ions (<3 V versus Li/Li^+^), which can lead to very high specific capacities (up to ∼580 mAh g^−1^_molecule_ and ∼493 mAh g^−1^_electrode_)^6^,^8^,^9^,^17^. However, these organic molecule-based electrodes suffer from low electrical conductivity and dissolution into the electrolyte, requiring mixing with a large amount of conductive carbon and polymer binders to increase electrical conductivity and to suppress dissolution^6^,^17^. Accordingly, improving the cycle stability and the rate capability without sacrificing the energy density of organic molecules remains a major challenge in the development of organic EES systems.

Here we report a new class of nanostructured organic electrodes for rechargeable EES systems, which are assembled by oxygen-functionalized FWNTs (few-walled carbon nanotubes) matrices and electropolymerized pyrene derivatives. These electrodes are pseudocapacitive in nature as the charge storage mechanism is based on redox reactions confined near the surface of the FWNTs and polymer materials. The electropolymerization of PAHs (polycyclic aromatic hydrocarbons) has previously been studied as potential conductive polymer films^24^,^25^,^26^,^27^. In addition, computational results have shown PAHs have the potential to protect graphite anodes in Li-ion batteries^28^. In this study, we investigate electrochemical reactions of various sized PAHs and identify pyrene, and its derivatives, as ideal candidates for use in organic EES systems, as they can be electropolymerized on various substrates, generating stable redox couples at high potentials over 3.5 V versus Li/Li^+^. Electropolymerized pyrene derivatives on an oxidized FWNT matrix have stable surface interactions with lithium ions and anions (PF_6_^−^), giving rise to a high specific capacity of ∼175 mAh g^−1^_electrode_. This results in an average capacitance value of 210 F g^−1^_electrode_. These organic free-standing electrodes exhibit a high energy density of ∼350 Wh kg^−1^_electrode_ and a high power density of ∼10 kW kg^−1^_electrode_ with little capacity degradation over 10,000 cycles (85% capacity retention). As a considerable amount of pyrene is available during the fractional distillation process of crude oil and coal tar^29^,^30^, nanostructured electrodes based on pyrene derivatives can be a promising strategy for the design of low-cost, efficient and reliable EES devices for large-scale applications.

## Results

### Screening of PAHs

**Figure 1:**
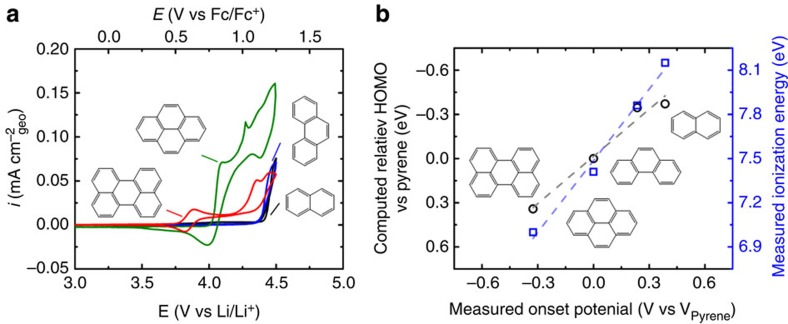
Screening of PAHs. (**a**) Cyclic voltammogram of first scan at 1 mV s^−1^ in 1 mM naphthalene (black), phenanthrene (blue), pyrene (green) and perylene (red) in solution. The redox potential of Fc/Fc^+^ was measured to be 3.25 V versus Li/Li^+^. (**b**) Computed relative HOMO energy level to that of pyrene and ionization energy^31^ as a function of measured oxidation onset potential in (**a**), referenced to that of pyrene. Dashed lines indicate the linear fit between calculated and experimental values. All molecules dissolved in EC:DMC (3:7 volume ratio) with 1 M LiPF_6_. Currents are normalized to the geometric area of the working electrodes.

The electrochemical properties of aromatic molecules of various sizes were studied in search of a material suitable for use as a positive electrode. Cyclic voltammetry (CV) scans of 1 mM solutions of naphthalene (two rings), phenanthrene (three rings), pyrene (four rings) and perylene (five rings) in a mixture of ethylene carbonate (EC) and dimethyl carbonate (DMC; 3:7 volume ratio) with 1 M LiPF_6_ within a three-electrode cell, employing a glassy carbon electrode (GCE) as the working electrode and lithium foil used as the counter and reference electrode, are shown in Fig. 1a. During the first forward scan, each molecule exhibited a distinct oxidation feature with different onset potentials. Naphthalene and phenanthrene showed relatively small oxidation currents on the forward scan at potentials over ∼4.3 V versus Li/Li^+^, while pyrene showed a large oxidation feature with an onset potential of ∼4.0 V versus Li/Li^+^, and perylene exhibited a reversible redox wave with E_1/2_ ∼3.8 V versus Li/Li^+^ and a large second oxidation feature with an onset potential of ∼4.2 V versus Li/Li^+^. The difference in the potentials of the CV profiles can be attributed to the different electronic structures of the molecules. The trend in the onset potential of oxidation was found to have a strong correlation with the highest occupied molecular orbital (HOMO) of each molecule, as well as the ionization energies as furnished from photoelectron spectroscopy^31^, as shown in Fig. 1b. As the polycyclic molecules increase in size, the HOMO energies increase, which is due to greater delocalization afforded by the π-system.

No reduction peaks were observed on the reverse scan in solutions of naphthalene and phenanthrene, suggesting a chemically irreversible event of the radical-cation intermediate, most likely arising from CC bonding^24^,^25^,^26^,^27^, with the ensuing product no longer being electrochemically active within the potential window of the CV scans. In contrast, a reduction peak centered around ∼4.0 V versus Li/Li^+^ was found for pyrene, while a reduction peak around ∼3.8 V versus Li/Li^+^ was observed for perylene in the reverse scan following the first oxidation event. On the other hand, the reduction currents of pyrene centered at ∼4 V versus Li/Li^+^ were much smaller than those on oxidation in the first cycle, indicative of a subsequent chemical reaction after oxidation. The formation of films on the GCE during CV cycling was found with pyrene in solution, but not for the other three molecules examined. There was little change in the CV profile on subsequent cycling for naphthalene, phenanthrene and perylene, as there was no change in the surface of the working electrode (Supplementary Fig. 1). Therefore, we hypothesize that pyrene has an optimum radical-cation stability for the polymerization reaction compared to those of other molecules. The film formation was confirmed by digital and helium ion microscope images (Supplementary Fig. 2 centre) and Raman and Fourier transform infrared spectra (Supplementary Fig. 3). To tune the redox potentials and morphologies of the polymerized films, we further investigated the effect of substituent groups on the redox process and polymerization of pyrene.

### Electrochemical properties of polymerized pyrene derivatives

**Figure 2:**
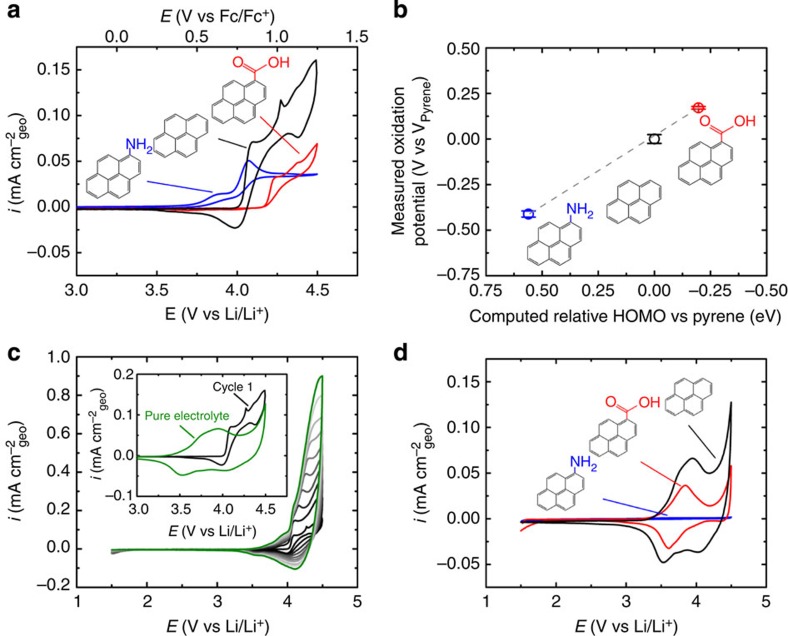
Electrochemical polymerization reaction of pyrene derivatives on a GCE. (**a**) The first CV scan at 1 mV s^−1^ of 1 mM aminopyrene (blue), pyrene (black) and pyrenecarboxylic acid (red) molecules in solution of EC:DMC (3:7 volume ratio) with 1 M LiPF_6_. (**b**) Measured change in oxidation onset potential of 1 mM of aminopyrene (blue), pyrene (black) and pyrenecarboxylic acid (red) molecules in solution from pyrene during CV at 1 mV s^−1^ as a function of the computed relative HOMO energy level to that of pyrene. Grey line indicates the linear fit between calculated and experimental values. (**c**) Cyclic voltammogram of polymerization reaction and growth of lower potential redox reaction during 10 cycles at 1 mV s^−1^ in 1 mM pyrene. (Inset) Overlap of initial polymerization cyclic voltammogram of pyrene and subsequent cyclic voltammogram of polymerized pyrene in pure electrolyte of 1 M LiPF_6_ in EC:DMC (3:7 volume ratio). (**d**) Cyclic voltammogram of polymerized aminopyrene (blue), pyrene (black) and pyrenecarboxylic acid (red) film during CV at 1 mV s^−1^ in 1 M LiPF_6_ in a mixture of EC and DMC (3:7 volume ratio).

CV scans of 1 mM solution of pyrene, pyrenecarboxylic acid and aminopyrene (pyrene derivatives) on GCEs, in a mixture of EC and DMC (3:7 volume ratio) with 1 M LiPF_6_ were performed to examine the effect of substituent groups on the redox behaviour of pyrene (Fig. 2a). Pyrenecarboxylic acid solutions demonstrated similar oxidation currents, but a higher onset potential of ∼4.1 V versus Li/Li^+^, consistent with the electron-withdrawing properties of the substituent. Conversely, the electron-donating properties of aminopyrene resulted in a lower onset potential of ∼3.6 V versus Li/Li^+^ and was the only molecule to demonstrate an oxidation peak, which occurred at ∼4.1 V versus Li/Li^+^, where the observation of a peak suggests diffusion limitations of the molecules to the GCE. The onset potential of each pyrene derivative was also measured at a concentration of 0.1 mM in a solution of EC and DMC (3:7 volume ratio) with 1 M LiPF_6_ (Supplementary Fig. 4), where similar shifts in onset potential relative to that of pyrene were found.

The onset oxidation potentials of pyrene derivatives with respect to that of pyrene were found to linearly correlate with the computed energy of the HOMO of pyrene derivatives referenced to that of pyrene (Fig. 2b), which is expected for a redox process accompanied by minimal structural reorganization^32^. In addition, the onset potential of the molecules show a linear correlation with the computed reduction potential of each molecule relative to that of pyrene and the Hammet substituent constant (Supplementary Fig. 5), where the electron-donating or -withdrawing tendency and resonance stabilization effects of a substituent group can be quantified by the Hammett substituent constant^33^ (0.45 for COOH and −0.66 for NH_2_)^34^. The onset oxidation potentials of pyrene were measured as a function of pyrene concentration in solution, showing that increasing the concentration of pyrene in solution led to a steady decrease in onset potential (Supplementary Fig. 6). The slope of the potential shift plotted on a log scale, as a function of concentration, was found to decrease by 28 mV per decade, which correlates well with the value expected from a two-electron transfer process per molecule (30 mV per decade) hypothesized to occur during the polymerization (inset of Supplementary Fig. 6a)^24^,^25^,^26^,^27^.

**Figure 3:**
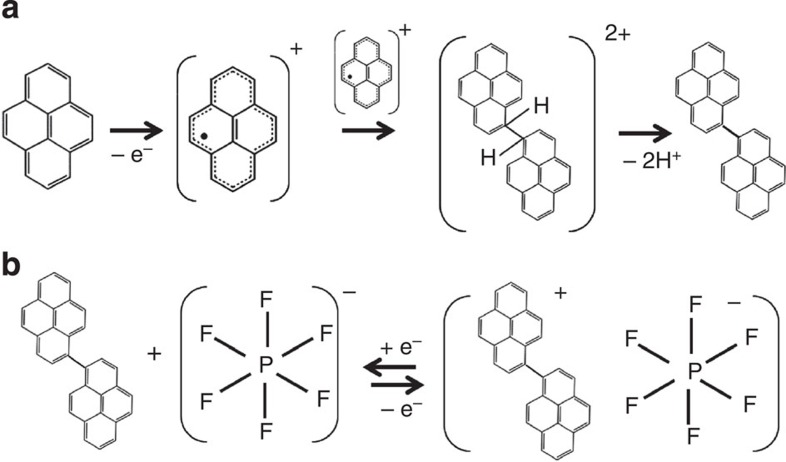
Proposed polymerization and reversible redox reactions. (**a**) Proposed polymerization reaction and (**b**) resulting reversible redox reaction of polymer film.

On subsequent potential cycling within the pyrene solution from 1.5 to 4.5 V versus Li/Li^+^, the oxidation current gradually increased and the onset potential of the oxidation feature gradually shifted to lower potentials, which is accompanied by the growth of reduction peaks (Fig. 2c). The oxidation currents can be ascribed to the oxidation of pyrene with simultaneous polymerization of the generated pyrene radical-cation intermediates on the GCE^24^,^25^,^26^,^27^. The electropolymerization of pyrene is believed to proceed through the deprotonation of ipso carbon atoms, as shown in Fig. 3a, which is supported by the decrease in pH of the solution after the polymerization^25^. The gradual decrease in the oxidation onset potential during the polymerization process can be related to the facilitated electrooxidation process due to the increased delocalization of π electrons in the longer π-motif, which is consistent with the trend in the electrooxidation of PAHs with increasing sizes, as shown in Fig. 1a (ref. 31). This hypothesis is further supported by the trend in calculated HOMO values of the pyrene dimer (0.15 eV versus HOMO of pyrene) and trimer (0.20 eV versus HOMO of pyrene) as the increase in the number of monomers correlates in an increase in the HOMO energy, resulting in a decrease in oxidation onset potential. On the other hand, the gradual appearance of peaks in the CV at 3.75 V versus Li/Li^+^ can be attributed to progressive anion (PF_6_^−^) doping process into the polymerized pyrene (Fig. 3b). The reversible redox wave of the polymerized pyrene film formed during 10 CV cycles was steady during subsequent CVs when the film was transferred to fresh electrolyte without the pyrene monomer (green line in the inset of Fig. 2c,d, Supplementary Fig. 7). The steady-state redox wave of the polymerized pyrenecarboxylic acid was similar to that of the polymerized pyrene, whereas the polymerized aminopyrene exhibited a much smaller current feature owing to minimal film formation (Fig. 2d and Supplementary Fig. 8).

Subsequent CV studies in different electrolytes support the occurrence of anion insertion into the polymerized pyrene films (Supplementary Fig. 9). The pyrene films, polymerized in the same electrolyte (LiPF_6_), were transferred to the electrolytes having lithium salts with different anions, including LiClO_4_, LiBF_4_ and LiPF_6_. The redox potentials of polymerized pyrene films in different electrolytes were found to increase from ClO_4_^−^, BF_4_^−^, to PF_6_^−^ (Supplementary Fig. 9), suggesting the anion is involved in the redox process and not the Li^+^ (Fig. 3b). Further support for the anion insertion mechanism came from electrochemical quartz crystal microbalance measurements, where the redox charge was correlated to mass variation (Supplementary Fig. 10). The steady increase in charge and mass from both film growth and anion incorporation was found during the oxidation process, whereas the decrease in charge and mass due to the removal of the anion was found during the reduction process. An average ratio of mass to charge passed of 141 g mol^−1^ was obtained for the species during reduction, with a s.d. of 43 g mol^−1^ for six independent measurements, and this average value is comparable to the molecular weight of PF_6_^−^ (145 g mol^−1^).

Potentiostatic oxidation of pyrene derivatives on indium tin oxide (ITO)-coated glass showed substantial film growth for pyrene and pyrenecarboxylic acid, but not for aminopyrene; these observations are consistent with the CV results (Fig. 2d and Supplementary Fig. 8). Potentiostatic oxidation of pyrene and pyrenecarboxylic acid solutions for 1 mAh cm^−2^_geo_ at 4.3 V versus Li/Li^+^ resulted in dark, thick coatings on ITO, which had thicknesses of 57 and 39 μm, respectively, whereas a yellow, thin coating on ITO (0.3 μm) was found for the aminopyrene solution (Supplementary Fig. 2). The thickness of polymerized pyrene was found to grow linearly and the corresponding root mean square roughness of the films increased with charge passed (Supplementary Fig. 11). As both the reactivity and stability of radical-cation intermediates are critical for polymerization^25^, it is proposed that the electron-donating amino group may reduce the reactivity of the radical-cation intermediates, preventing the propagation reaction of the radical aminopyrene to large chains of polymerized aminopyrene on the surface. Thus, most of the current passed may be used to form soluble species such as dimers, trimers, and oligomers, which can diffuse into the solution instead of participating in the formation of a polymer film on the electrode.

### Polymerized pyrene derivatives on FWNTs

**Figure 4:**
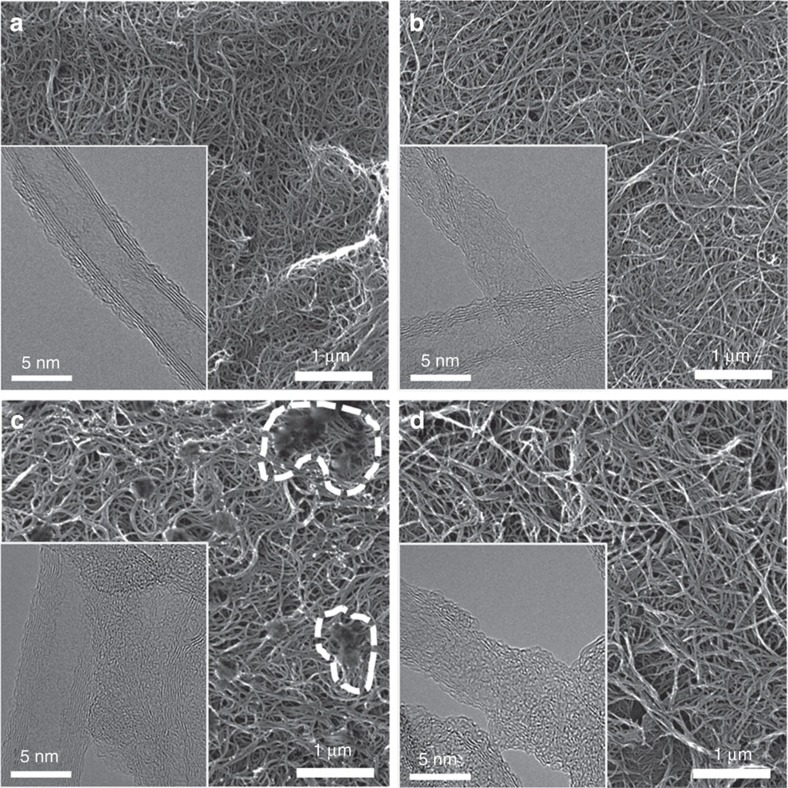
Electrochemical polymerization of pyrene derivatives on FWNT substrate. Helium ion microscope images of (**a**) FWNT, (**b**) oxidized FWNT, (**c**) FWNT/pyrenecarboxylic acid and (**d**) oxidized FWNT/aminopyrene electrodes. (Inset) HRTEM images of the same type of electrode.

Free-standing electrodes of pristine and oxidized FWNTs, which were assembled by vacuum filtration^35^, were used as substrates to polymerize pyrene derivatives in two-electrode cells with lithium negative electrodes. The conducting FWNT substrates have a large specific surface area 400 m^2^ g^−1^, which can incorporate the large amounts of polymerized pyrene derivatives into the electrodes. Pristine and oxidized FWNT electrodes both showed interpenetrating network structures and the exterior walls become roughened during the oxidation process for the oxidized FWNTs (Fig. 4a,b). Polymerization of pyrene derivatives on pristine FWNT and oxidized FWNT electrodes were conducted in a two-electrode cell with a lithium negative electrode using a CV cycling process (Supplementary Figs 12 and 13). The polymer film formation was confirmed by X-ray photoelectron spectroscopy analysis (Supplementary Fig. 14 and Supplementary Table 1). Polymerized pyrene, pyrenecarboxylic acid and aminopyrene on pristine FWNTs showed similar inhomogeneous coatings on FWNTs (Fig. 4c and Supplementary Fig. 15a,b). For example, the polymerized coating of pyrenecarboxylic acid on pristine FWNTs formed micron-scale agglomerates (circled in Fig. 4c and shown in Fig. 4c inset). Polymerized pyrene on oxidized FWNTs was found to fill the entire void area, while polymerized pyrenecarboxylic acid was found to form an irregular coating on the oxidized FWNTs (Supplementary Fig. 15c,d). In contrast, polymerization of aminopyrene on oxidized FWNTs led to the most conformal coating on individual oxidized FWNTs without the formation of micrometre-scale granules (Fig. 4d and inset). The formation of a conformal coating on oxidized FWNTs from oxidation of aminopyrene in solution may be facilitated by the strong electrostatic interaction between negatively charged oxygen functional groups (for example, –COO^−^) on oxidized FWNTs and protonated amino groups (−NH_3_^+^) on aminopyrene in contrast to the weak π–π interaction between pristine FWNTs and pyrene derivatives. It is speculated that this strong electrostatic interaction may trap monomers or other soluble species on the FWNT surface and result in the uniform polymerization.

**Figure 5:**
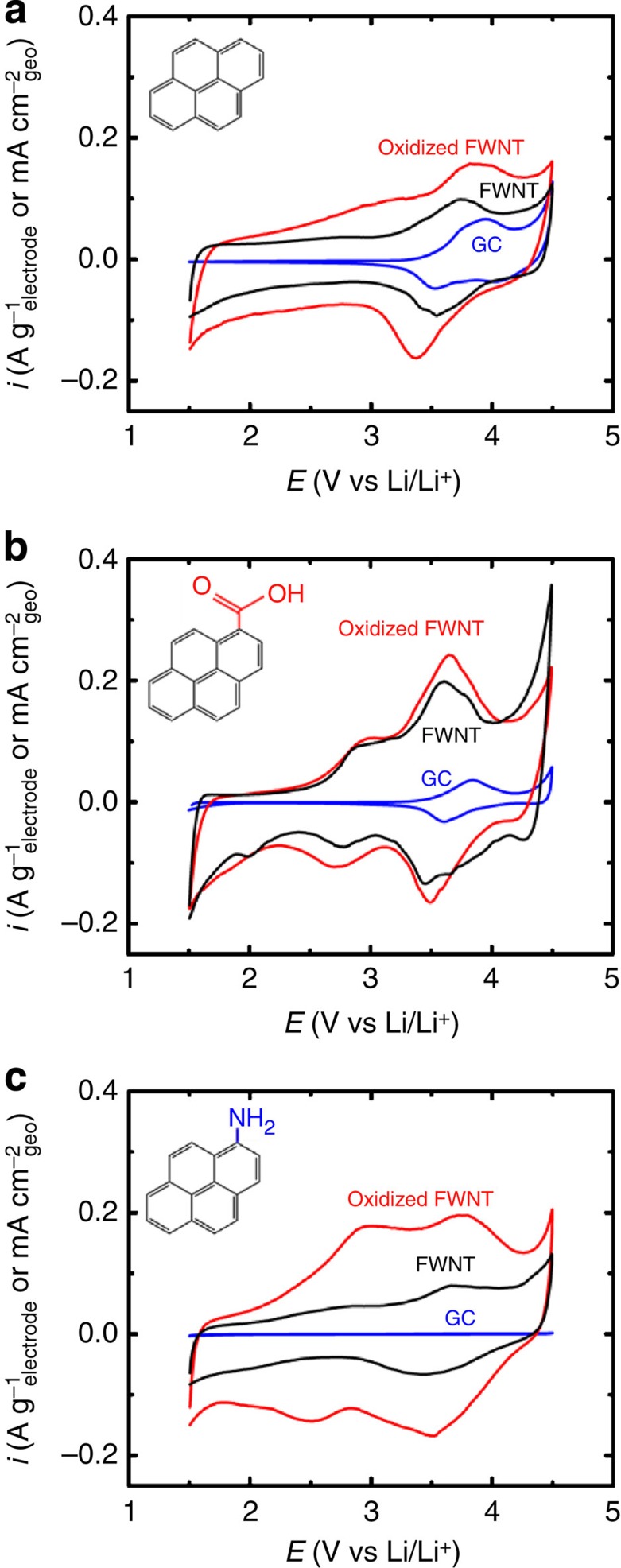
Redox reactions of polymerized pyrene derivatives on various substrates. Steady-state cyclic voltammograms of polymerized (**a**) pyrene (41 wt% FWNT and 34 wt% oxidized FWNT), (**b**) pyrenecarboxylic acid (28 wt% FWNT and 34 wt% oxidized FWNT), (**c**) aminopyrene (58 wt% FWNT and 43 wt% oxidized FWNT) on a GCE (blue), FWNT substrate (black) and oxidized FWNT substrate (red) between 1.5–4.5 V versus Li/Li^+^ at a scan rate of 1 mV s^−1^ in EC:DMC (3:7 volume ratio) with 1 M LiPF_6_ with no monomer in solution. Vertical scale units are A g^−1^_electrode_ for FWNT and Oxidized FWNT electrodes and mA cm^−2^_geo_ for GCE. Note that the specific surface area of the FWNT substrate is ∼400 m^2^ g^−1^.

The surface redox reactions of pyrene derivatives on the different substrates were investigated through CV. Steady-state redox reactions during CV scans of polymerized pyrene derivatives on GCEs, pristine FWNTs and oxidized FWNTs are compared in Fig. 5. Polymerized pyrene derivatives on pristine and oxidized FWNTs showed pronounced redox peaks at ∼3.7 V versus Li/Li^+^ from anion interactions with the polymer, similar to those on GCEs (Fig. 5a). In addition to this redox process, polymerized pyrenecarboxylic acid and aminopyrene on oxidized FWNTs exhibited additional redox events at ∼3 V versus Li/Li^+^, which was not found on GCEs (Fig. 5b,c and Supplementary Fig. 8). The currents associated with the process at ∼3 V versus Li/Li^+^ are the most significant for polymerized aminopyrene on oxidized FWNTs (Fig. 5c). It is proposed that the additional redox process at ∼3 V versus Li/Li^+^ can be attributed to lithium ion interaction with oxygen-containing functional groups^36^ on the oxidized FWNTs^35^ or the carboxylic groups on the polymerized pyrenecarboxylic acid, as similar redox peaks have been reported for aromatic carbonyl derivatives, such as poly(2,5-dihydroxy-1,4-benzoquinone-3,6-methylene)^37^ and Li_2_C_6_O_6_ (ref. 8). In addition, the enhanced redox behaviour on three-dimensional FWNTs compared with two-dimensional GCEs show that the nanostructured conducting substrate can support the fast surface redox process taking place on the polymerized pyrene derivatives.

**Figure 6:**
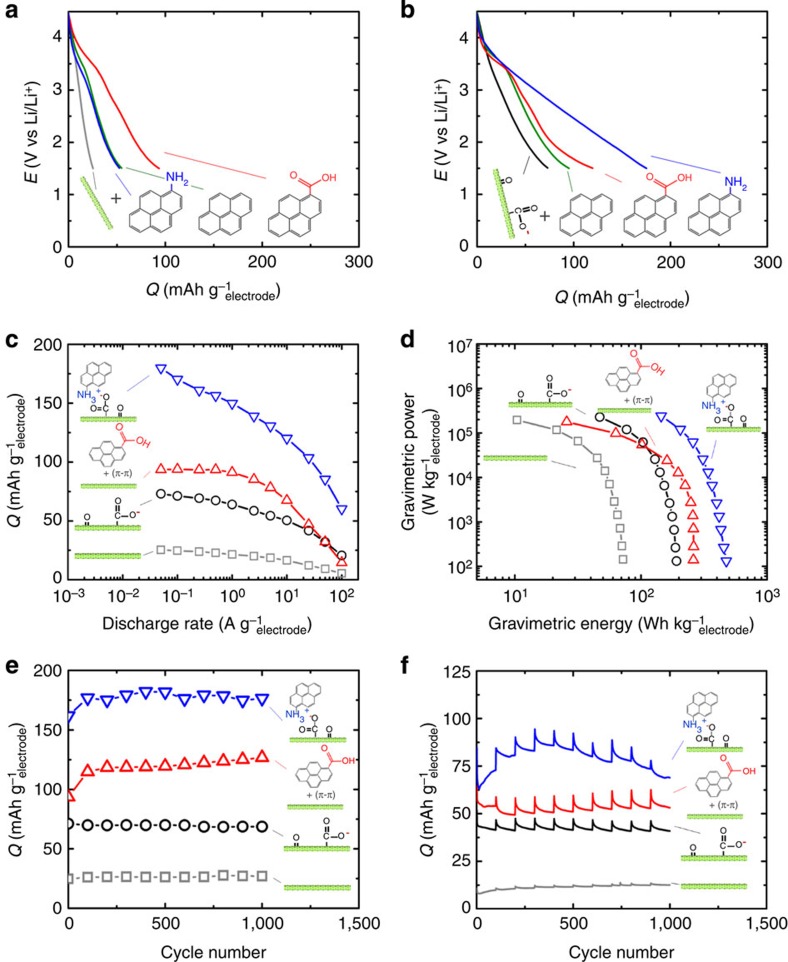
Electrochemical performance of polymerized pyrene derivatives within FWNT substrate. (**a**) Discharge profiles of pristine FWNT (grey), FWNT/polypyrene (41 wt% FWNT) (green), FWNT/polypyrenecarboxylic acid (28 wt% FWNT; red) and FWNT/polyaminopyrene (58 wt% FWNT; blue) electrodes at a discharge current density of 0.05 A g^−1^. (**b**) Discharge profiles of oxidized FWNTs (black), oxidized FWNT/polypyrene (34 wt% FWNT; green), oxidized FWNT/polypyrenecarboxylic acid (34 wt% FWNT; red) and oxidized FWNT/polyaminopyrene (43 wt% FWNT; blue) electrodes at a discharge current density of 0.05 A g^−1^. (**c**) Specific capacities as a function of discharge rate for FWNTs (grey), oxidized FWNTs (black), FWNT/polypyrenecarboxylic acid (28 wt% FWNT; red) and oxidized FWNT/polyaminopyrene (66 wt% FWNT; blue) electrodes. (**d**) Ragone plot comparing energy and power densities of pristine FWNT (grey), oxidized FWNT (black), FWNT/pyrenecarboxylic acid (28 wt% FWNT; red) and oxidized FWNT/aminopyrene (66 wt% FWNT; blue) electrodes with a lithium negative electrode. (**e**) Specific capacities of FWNTs (grey), oxidized FWNTs (black), FWNT/polypyrenecarboxylic acid (28 wt% FWNT; red) and oxidized FWNT/polyaminopyrene (43 wt% FWNT; blue) electrodes as a function of cycle number, which were measured at a current density of 0.1 A g^−1^ once every 100 cycles after voltage holds at the end of charge and discharge for 30 min. Within each 100 cycles, these cells were cycled under a rate of 10 A g^−1^. (**f**) Specific capacities of electrodes in (**e**) as a function of cycle number for the intermediate cycles at a rate of 10 A g^−1^. We speculate the initial capacity increase comes from the electrolyte having additional time to diffuse into the pores of the electrode, allowing access to additional active material. In addition, the capacity increase can be attributed to the continued polymerization of monomers remaining on the surface of the electrode and in the electrolyte. The high-rate specific capacities show hysteresis from the slow discharge cycles once every 100 cycles. The thickness and density of the electrodes range from 12 to 65 μm and 0.2 to 0.7 g cm^−3^, respectively. The density for oxidized FWNT/aminopyrene is ∼0.6 g cm^−3^. The weight of the total positive electrode (pyrene derivative+FWNT electrode) was considered in the energy and power density calculations.

The surface redox reactions with polymerized pyrene derivatives on FWNTs enable the composite electrodes to have high specific capacities. The specific capacity and rate capability of polymerized pyrene derivatives on FWNTs were evaluated using galvanostatic tests in the range of 1.5–4.5 V versus Li/Li^+^ (Fig. 6a,b and Supplementary Figs 16 and 17). The specific capacities (FWNT plus pyrene derivatives, Supplementary Fig. 16) were found to increase from ∼25 mAh g^−1^_electrode_ (average capacitance of 30 F g^−1^_electrode_) to ∼50 mAh g^−1^_electrode_ (average capacitance of 60 F g^−1^_electrode_) for polymerized pyrene and to ∼94 mAh g^−1^_electrode_ (average capacitance of 113 F g^−1^_electrode_) for polymerized pyrenecarboxylic acid on pristine FWNTs, which is in good agreement with CV measurements shown in Fig. 5. The electrode capacities were further increased to ∼175 mAh g^−1^_electrode_ for polymerized aminopyrene on oxidized FWNTs, as shown in Fig. 6b. This results in an average capacitance value of 210 F g^−1^_electrode_. This large capacity can be attributed to two redox processes at ∼2.9 V (lithium-ion incorporation) and at ∼3.6 V (anion incorporation) versus Li/Li^+^ (Supplementary Fig. 18), and conformal coating of polymerized aminopyrene on oxidized FWNTs, which could allow higher electrochemical utilization of polymerized aminopyrene on oxidized FWNTs, relative to polymerized pyrene and pyrene carboxylic acid on oxidized FWNTs with irregular micrometre-scale polymer agglomerates. The specific capacities as a function of discharge rate are provided in Fig. 6c. Polymerized aminopyrene on oxidized FWNTs can sustain ∼70% of the capacity over two orders of magnitude of discharge rate from 0.1 to 10 A g^−1^. Capacity retention for polymerized pyrenecarboxylic acid on FWNTs, oxidized FWNTs and pristine FWNTs are similar to that of polymerized aminopyrene on oxidized FWNTs, as their capacity at 10 A g^−1^ is ∼70% of that at 0.1 A g^−1^. In addition, the coulombic efficiencies at various discharge rates are close to 100% for the polymerized aminopyrene on oxidized FWNTs electrode (Supplementary Fig. 19).

To access the potential for these materials to be used for EES devices, the energy and power densities of pristine and oxidized FWNTs and FWNTs with polymerized pyrene derivatives in two-electrode cells with lithium negative electrodes are compared. Results from the comparison are shown in Fig. 6d (other electrodes in Supplementary Figs 20 and 21). Incorporation of polymerized aminopyrene on oxidized FWNTs significantly increased the gravimetric energy density of FWNT electrodes without sacrificing power density. At a low power (∼100 W kg^−1^_electrode_), polymerized aminopyrene on oxidized FWNTs exhibited a high energy density of ∼475 Wh kg^−1^_electrode_. This energy density is significantly higher compared with those (300–350 Wh kg^−1^_electrode_) of the oxidized FWNT and oxidized multi-wall CNT/graphene composite electrodes assembled by vacuum filtration process^35^,^38^ and also even higher than that (∼420 Wh kg^−1^_electrode_) of 3-μm-thick thin-film CNT electrodes assembled by a layer-by-layer process^36^. Polymerized aminopyrene on oxidized FWNTs can deliver an energy density of ∼350 Wh kg^−1^_electrode_ at a high power of ∼10 kW kg^−1^_electrode_. This corresponds to a volumetric energy density of ∼195 Wh L^−1^_electrode_ at a volumetric power density of ∼5.6 kW L^−1^_electrode_ (Supplementary Fig. 22). Moreover, minimal capacity loss was found in the specific capacities of these electrodes up to 1,000 cycles (Fig. 6e) and extended cycling up to 11,000 cycles showed small capacity loss ∼15% for polymerized aminopyrene on oxidized FWNTs (Supplementary Fig. 23a). Initial capacity increase of the FWNT/polymerized pyrene derivative electrodes was found, which can be attributed to the enhanced electrolyte accessibility into redox active materials and to the continued polymerization of monomers remaining on the surface of the electrodes and in the electrolyte. Cycling tests consisted of high-rate cycles at 10 A g^−1^ for 99 cycles, followed by a slower-rate cycle at 0.1 A g^−1^ every 100 cycles up to 1,000 cycles. The specific capacities during the high-rate discharge cycles at 10 A g^−1^ are shown in Fig. 6f, where there is again minimal specific capacity loss. The recovery in specific capacity every 100 cycles occurs after the slow-rate discharge cycle at 0.1 A g^−1^. The Supplementary Fig. 23b displays the extended high-rate cycles for polymerized aminopyrene on oxidized FWNTs up to 3,000 cycles with negligible capacity loss, confirming the stability of the electrode during the high-rate cycle condition. The cycling stability of polymerized aminopyrene on oxidized FWNTs may be attributed to the nanoscale conformal coatings of polymers mediated by the interactions between surface functionalities between polymers and oxidized FWNTs. These values presented are the optimal values for these positive electrodes as a negative electrode other than metallic lithium will need to be used in practice. This approach demonstrates a new design concept of organic electrodes based on the surface functionalities, which exhibits high energy and power density and superior cycling stability compared to other nanostructured organic electrodes^6^,^8^,^9^,^16^,^17^,^18^,^19^,^20^,^39^,^40^,^41^,^42^,^43^,^44^,^45^,^46^,^47^.

## Discussion

We have fabricated high-energy and high-power nanostructured organic electrodes utilizing surface redox reactions of polymerized pyrene derivatives on the functionalized FWNTs. Electropolymerization and redox properties of pyrene derivatives were correlated with the substituent groups and their electronic structure on the planar substrates. Pyrene derivatives were conjugated to functionalized FWNT substrates to maximize the electrochemical reactions based on the unit mass of the electrodes. By using the electrostatic interaction between oxygen functional groups and protonated amino groups, an optimized nanostructured electrode was designed. Using surface redox reactions, the oxidized FWNT/aminopyrene electrode delivered a high gravimetric capacity of ∼175 mAh g^−1^_electrode_. The oxidized FWNT/aminopyrene electrode has a high energy density of ∼475 Wh kg^−1^_electrode_ at low power, and maintains an energy density of ∼350 Wh kg^−1^_electrode_ at a power density of ∼10 kW kg^−1^_electrode_. The FWNT with pyrene derivative electrodes are self-standing and free of inactive binders and current collectors, enhancing the energy and power density of practical devices. In addition, polymerized pyrene derivatives can be incorporated into other high surface area carbon materials, including reduced graphene oxides. Large-scale energy-storage applications based on pyrene is facilitated by their availability from the fractional distillation of crude oil and coal tar^29^,^30^. The dispersion of polymerized pyrene or perylene derivatives within aqueous or non-aqueous electrolytes may allow for the fabrication of organic redox flow batteries^48^,^49^,^50^ for large-scale applications. The results highlight that earth abundant organic materials can be integrated into functional substrates and act as high-performance nanostructured electrodes for future energy-storage applications.

## Methods

### Electrochemical measurements

All experiments on glassy carbon (GCE, CH Instrument Inc.) and ITO working electrodes (Rs=4–8 Ω, Delta Technologies, LTD) were carried out in a three-electrode cell at room temperature using a Biologic (SP300 or VSP300) potentiostat, lithium as the counter electrode and lithium as the reference electrode. Electrochemical quartz crystal microbalance measurements used a Au-coated quartz crystal working electrode and cell from Biologic, in conjunction with a Seiko quartz crystal analyser and a lithium counter electrode. Naphthalene (99.7%, Fluka), phenanthrene (99.5%, Aldrich), pyrene (99%, Aldrich) and perylene (99.5%, Aldrich) were dispersed in the stated concentrations within EC:DMC (3:7 volume ratio) with 1 M LiPF_6_ or 1 M LiBF_4_ (<20 p.p.m. H_2_O, BASF) or was used within EC:DMC (3:7 volume ratio) with dried 1 M LiClO_4_ (99.99%, Aldrich).

All experiments on FWNT electrodes were done in two-electrode cells (Tomcell, Japan) assembled inside an argon-filled glovebox (Vacuum Atmosphere Co., O_2_ level below 1 p.p.m. and H_2_O level below 1 p.p.m.). The cell consisted of a free-standing vacuum filtration-FWNT positive electrode, a lithium negative electrode, two porous Celgard 2500 separators and 1 M LiPF_6_ in an EC:DMC (3:7 volume ratio) electrolyte. The electrodes were tested electrochemically using a Solartron 1470 test unit in the voltage range 1.5–4.5 V versus Li/Li^+^. For galvanostatic rate capability tests, the current densities ranged from 0.05 to 100 A g^−1^, corresponding to C-rates from 0.29 C to 580 C in the case of the oxidized FWNT/aminopyrene electrode. The voltage was held constant at either 1.5 or 4.5 V versus Li/Li^+^ for 30 min before charge or discharge, respectively. Galvanostatic capacities can vary depending on the amount and morphology of polymer coating. For example, the case with the largest variability—the oxidized FWNT/aminopyrene electrode—the capacity can range from ∼100 to 180 mAh g^−1^_electrode_ depending on the amount of polymer coating. The loading of the polymer coating has been found to vary from ∼21 to 63% of the total weight of the electrode. Cycling tests consisted of galvanostatic cycling at 10 A g^−1^ for 99 cycles, followed by a slower charge and discharge cycle at 0.1 A g^−1^ every 100 cycles up to 1,000 cycles, with a 30-min voltage hold at 1.5 V or 4.5 V versus Li/Li^+^ before low-rate charge or discharge, respectively. For cycle numbers between 1,000 and 11,000, 499 accelerated cycles were performed for every slower charge and discharge cycle.

All performance metrics were normalized to the total weight of the positive electrode, including both the FWNT and polymer coating. Energy densities are calculated based on the integration of the discharge profiles of the potential as a function of specific capacity. The average power density during discharge is reported on all Ragone plots. Additional details on the methods of calculating performance metrics are provided in the Supplementary Methods.

### Sample preparation

Sub-millimetre long few-walled nanotubes (FWNTs; 6–10 nm diameter, 0.4 mm length, 99 wt% purity, 400 m^2^ g^−1^ specific surface area, triple walled on average) were synthesized by chemical vapour deposition in a single fluidized bed reactor^51^.

Functionalization of FWNT was performed using a previously reported method^35^. In brief, functionalized FWNTs were prepared by oxidizing pristine FWNTs in a mixture of H_2_SO_4_ (96.5%, Aldrich) and HNO_3_ (70%, Aldrich) solution (3:1 volume ratio) at 70 °C for 2 h. The functionalized FWNTs were washed in a ∼5% by volume HCl solution and dried in air.

FWNTs or oxidized FWNTs were mixed with stated molecules (pyrene (99%, Aldrich), 1-aminopyrene (97%, Aldrich), 1-pyrenecarboxylic acid (97%, Aldrich)) in various ratios, dispersed in Milli-Q deionized water and ethanol at a concentration of 0.2 mg ml^−1^. Binder-free electrodes were synthesized through vacuum filtration on Celgard 2500 membranes (64 nm average pore diameter). Electrodes were further dried at 70 °C under vacuum and polymerized in a two-electrode cell typically through five CV cycles at 1 mV s^−1^ and subsequent five CV cycles at 5 mV s^−1^ between 1.5–4.5 V versus Li/Li^+^.

### Characterization

Microstructure of polymer electrodes were investigated using a scanning helium ion microscope (Orion Plus Helium Ion Microscope from Carl Zeiss) operating at 30 kVs and a JEOL 2010 transmission electron microscope with a LaB_6_ thermal emission electron gun was used for high-resolution imaging. The microscope was operated at 200 kV and was able to ultimately achieve 0.19 nm point-to-point resolution.

For identifying the surface chemistry of the polymer electrodes, a Physical Electronics Versaprobe II X-ray Photoelectron Spectrometer was used. The relative sensitivity factors used to scale the peaks of C 1s, N 1S, O 1s, F 1s and P 2p were 58.791, 93.486, 137.408, 187.576 and 113.106, respectively.

The thickness of the polymer coated on the ITO substrate was determined by scratching away the polymer to the substrate and averaging the thickness at four locations across the step change in height using a Tencor P-16 Surface profilometer. The densities of the electrodes were determined by measuring the mass and volume of the polymer and FWNT substrate. The volume of each FWNT electrode was determined by multiplying the thickness and the geometric area of the electrode.

### Additional methods

Additional details on the experimental and computational methods can be found within the Supplementary Methods section within the Supplementary Information.

## Author contributions

Y.S.-H., S.W.L., J.C.B., R.K. and D.G.N. conceived and designed the experiments. D.Y.K. and S.N. contributed to methods of few-walled carbon nanotubes film synthesis. D.J.G. contributed to computational analysis. Y.S.H., S.W.L., J.C.B., and D.G.N. co-wrote the manuscript.

## Additional information

**How to cite this article:** Bachman, J. C. *et al.* Electrochemical polymerization of pyrene derivatives on functionalized carbon nanotubes for pseudocapacitive electrodes. *Nat. Commun.* 6:7040 doi: 10.1038/ncomms8040 (2015).

## Supplementary Material

Supplementary Information Supplementary Figures 1-24, Supplementary Table 1, Supplementary Methods and Supplementary References
