# Supplementary material for: Electrochemical polymerization of pyrene derivatives on functionalized carbon nanotubes for pseudocapacitive electrodes
Source: Nat Commun. 2015 May 6;6:7040. doi: 10.1038/ncomms8040 (PMC4432658; doi:10.1038/ncomms8040)
Supplement: Supplementary Information — Supplementary Figures 1-24, Supplementary Table 1, Supplementary Methods and Supplementary References [file ncomms8040-s1.pdf]

Supplementary Figures

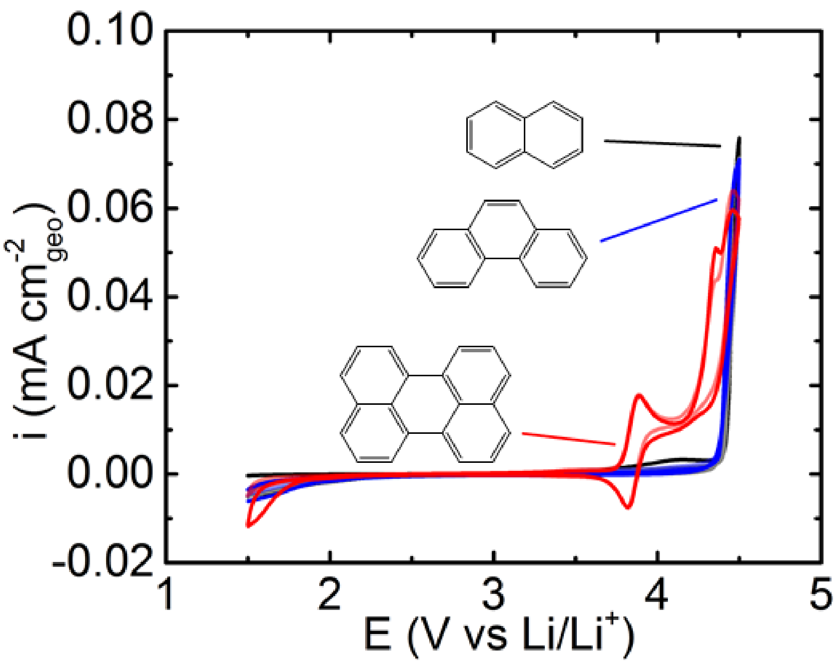

Figure 1. Cyclic voltammogram of first scan (darker color) and fifth scan (lighter color) at 1 mV s^{-1} in 1 mM naphthalene (black), phenanthrene (blue), and perylene (red) in solution. All molecules dissolved in EC:DMC (3:7 volume ratio) with 1 M $LiPF_6$. Currents are normalized to the geometric area of the working electrode.

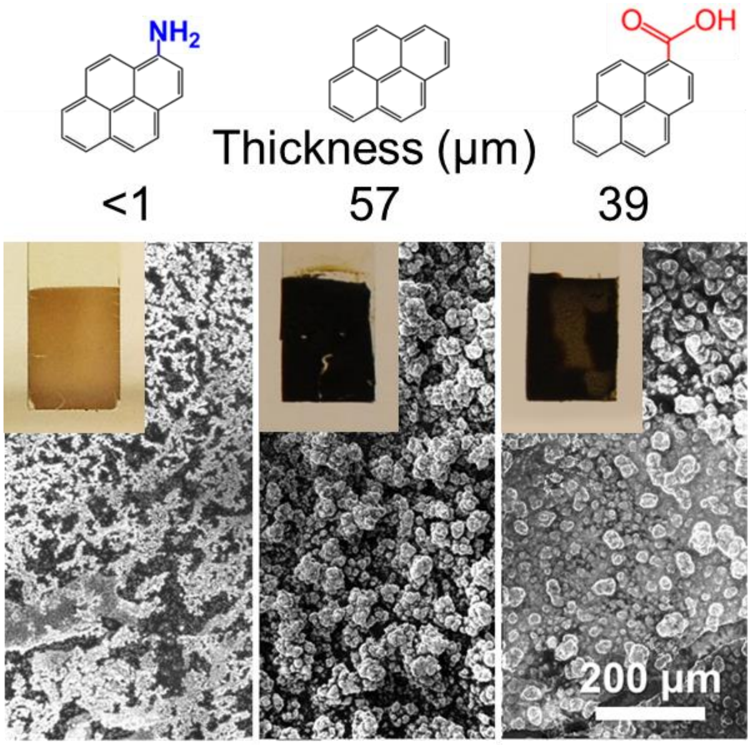

Figure 2. Digital and helium ion microscope images of polymerized pyrene derivatives on ITO coated glass slides (ITO held at 4.3 V versus Li/Li^+ for 1 mAh cm^{-2} in solution with 1 mM pyrene derivative). Film thickness are shown above images. All molecules dissolved in EC:DMC (3:7 volume ratio) with 1 M $LiPF_6$ for polymerization.

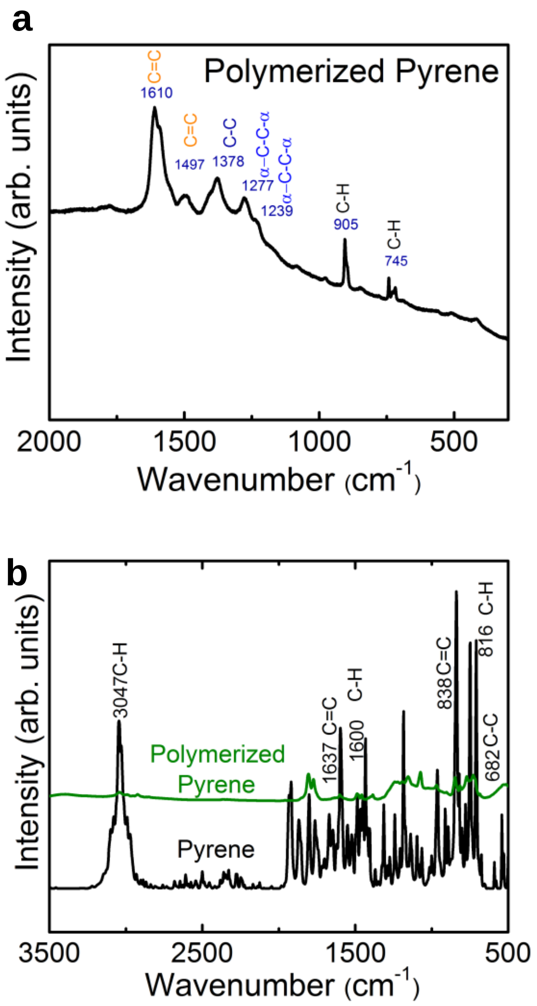

Figure 3. (a) Raman spectra of polymerized pyrene on Au electrode after potentiostatic elctropolymerization at 4.3 V versus Li/Li^+ in 1 M $LiPF_6$ and 1 mM pyrene in a mixture of EC and DMC (3:7 volume ratio). The Raman spectra shows a peak at ~1610 cm^{-1}, which can be assigned to the stretching mode of the C=C bond. The strong peak at 1378 cm^{-1} belongs to the C-C interring stretching. The peaks close to 1277 and 1239 cm^{-1} correspond to the α-C-C-α stretching vibration between two neighboring pyrene molecules. The 1142 cm^{-1} band is associated with the symmetric in-plane vibration of C-H bonds. The peaks at 905 and 745 cm^{-1} are related to the C-H deformation.[8] **(b)** Fourier transform infrared spectra of the same polymerized pyrene film.

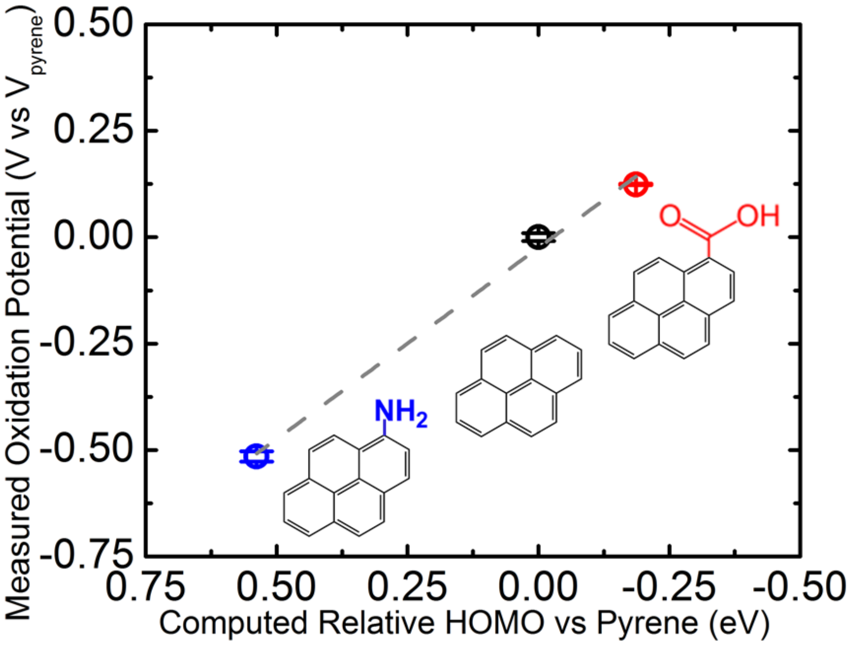

Figure 4. Measured oxidation onset potential of 0.1 mM aminopyrene (blue), pyrene (black), and pyrenecarboxylic acid (red) molecules during cyclic voltammetry at 1 mV s^{-1} in 1 M LiPF6 in a mixture of EC and DMC (3:7 volume ratio) as a function of the computed relative HOMO energy to that of pyrene. Error bars represent standard deviation of three measurements.

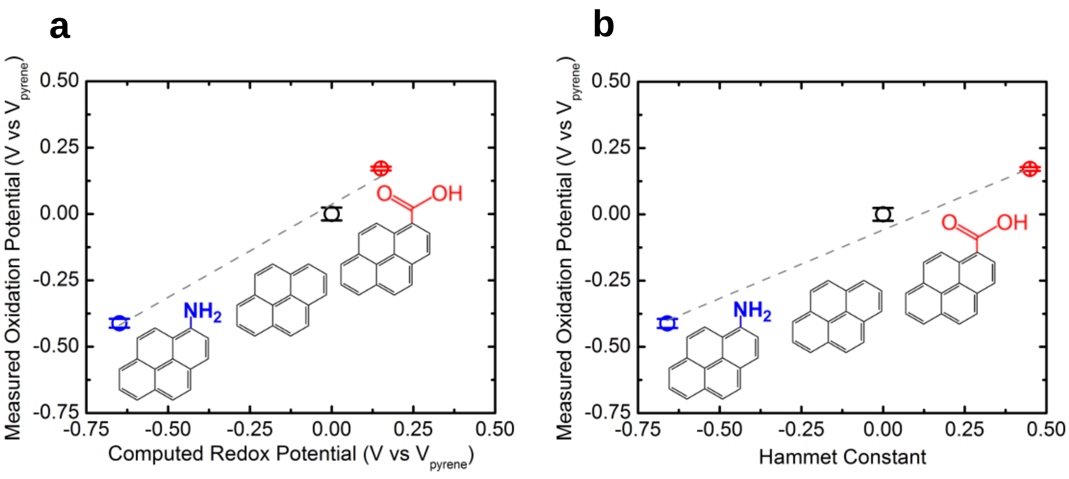

Figure 5. Measured oxidation onset potential of 1 mM aminopyrene (blue), pyrene (black), and pyrenecarboxylic acid (red) molecules during cyclic voltammetry at 1 mV s^{-1} in 1 M LiPF6 in a mixture of EC and DMC (3:7 volume ratio) **(a)** as a function of the computed relative redox potential versus that of pyrene and **(b)** as a function of the Hammet constant. Gray lines indicate the linear fit of the data. Error bars represent standard deviation of three measurements.

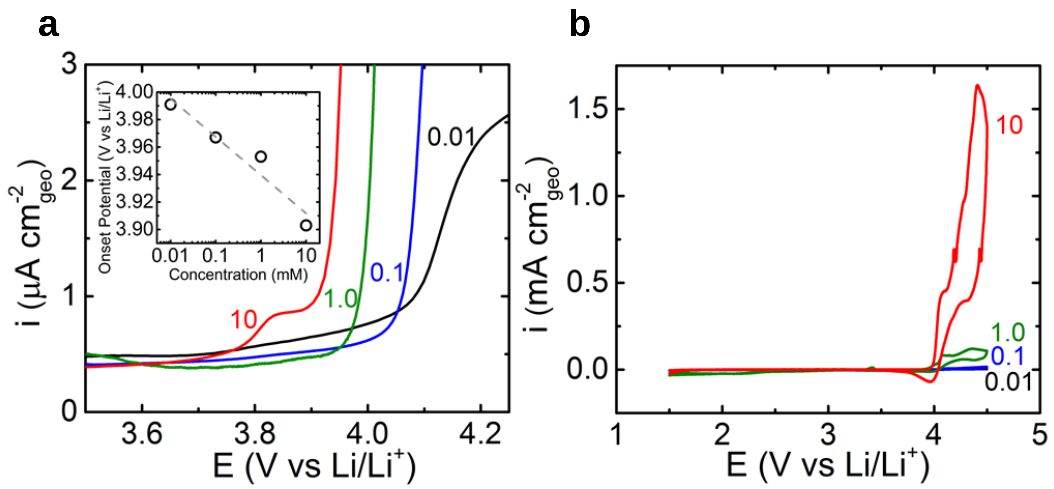

Figure 6. (a) First forward cyclic voltammetry scan at 1 mV s^{-1} with pyrene in solution at various concentrations in 1 M $LiPF_6$ in a mixture of EC and DMC (3:7 volume ratio). Pyrene concentrations are 0.01 (black), 0.1 (blue), 1.0 (green), and 10 mM (red). **(Inset)** Measured onset potential as a function of the concentration of pyrene in solution. **(b)** Full cyclic voltammogram of scans in (a).

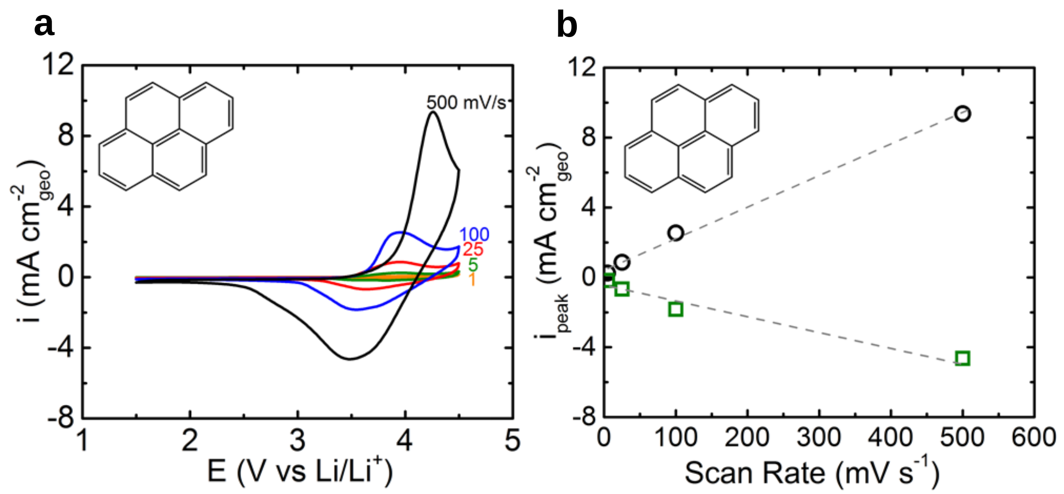

Figure 7. (a) Scan-rate dependent CV comparison of polymerized pyrene over a range of scan rates in 1 M $LiPF_6$ in a mixture of EC and DMC (3:7 volume ratio). **(b)** Peak current as a function of a scan rate.

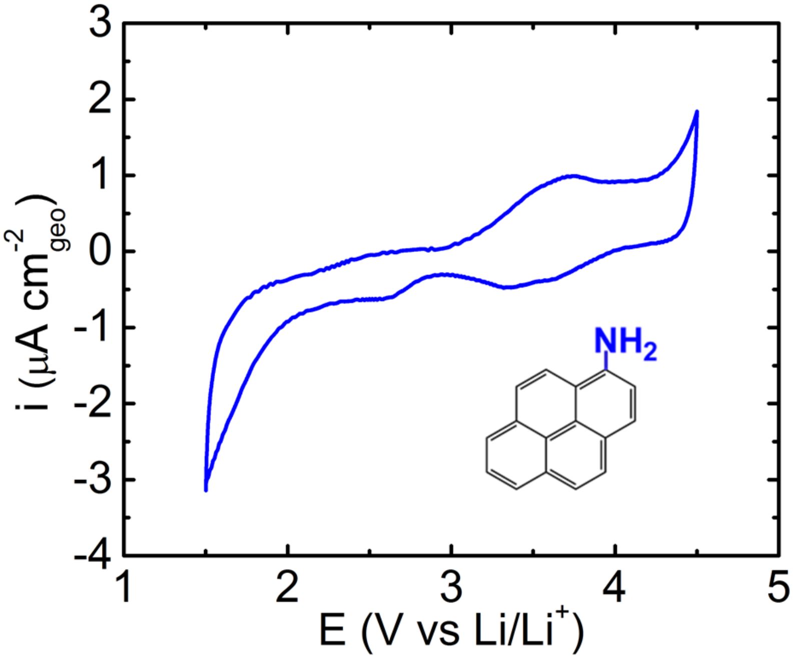

Figure 8. Cyclic voltammogram of polymerized aminopyrene film during cyclic voltammetry at 1 mV s^{-1} in 1 M $LiPF_6$ in a mixture of EC and DMC (3:7 volume ratio).

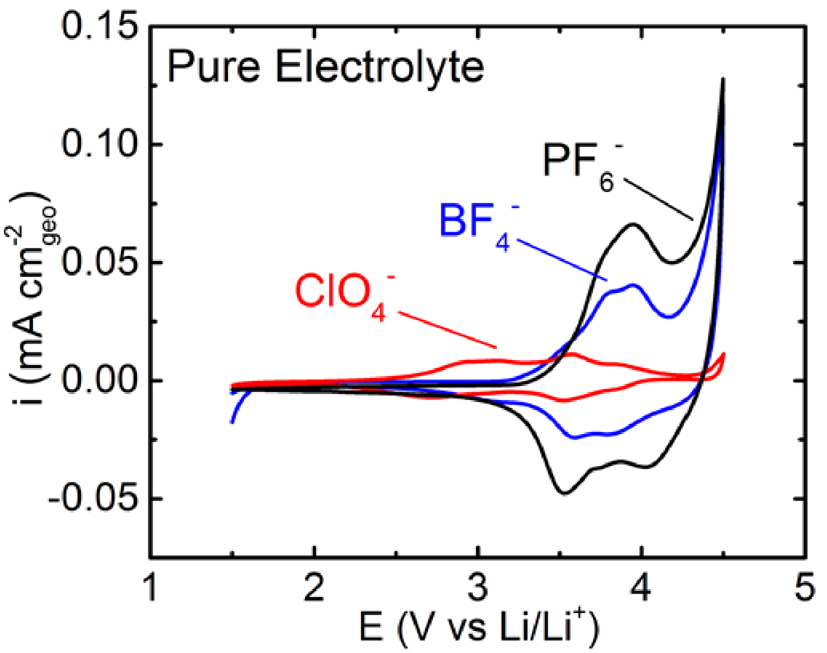

Figure 9. Redox reaction of polymerized pyrene films during cyclic voltammetry at 1 mV s^{-1} with 1 M $LiClO_4$ (red), $LiBF_4$ (blue), and $LiPF_6$ (black) grown under the conditions of Figure 2C.

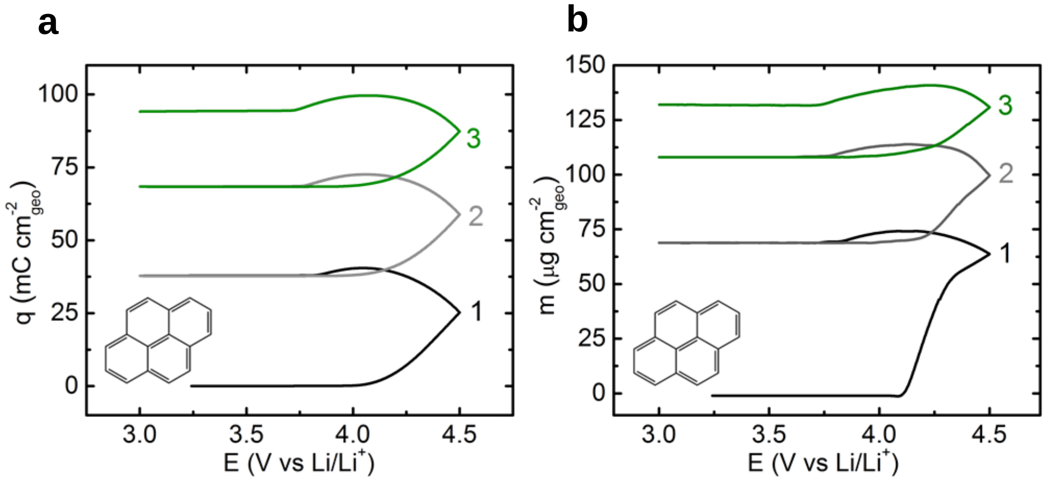

Figure 10. (a) Charge passed during three cycles at 1 mV s^{-1} in 10 mM pyrene in EC:DMC (3:7 volume ratio) with 1 M $LiPF_6$ on a Au electrode, while simultaneously measuring the change in weight on the electrode with an electrochemical quartz crystal microbalance. **(b)** Resulting mass on the Au electrode during cycling of (a). When calculating the ratio of mass to charge for the six total reduction processes, an average value of 141 g mol^{-1} was obtained (with a standard deviation of 43 g mol^{-1}). This average value is very similar to 145 g mol^{-1} of PF_6^-.

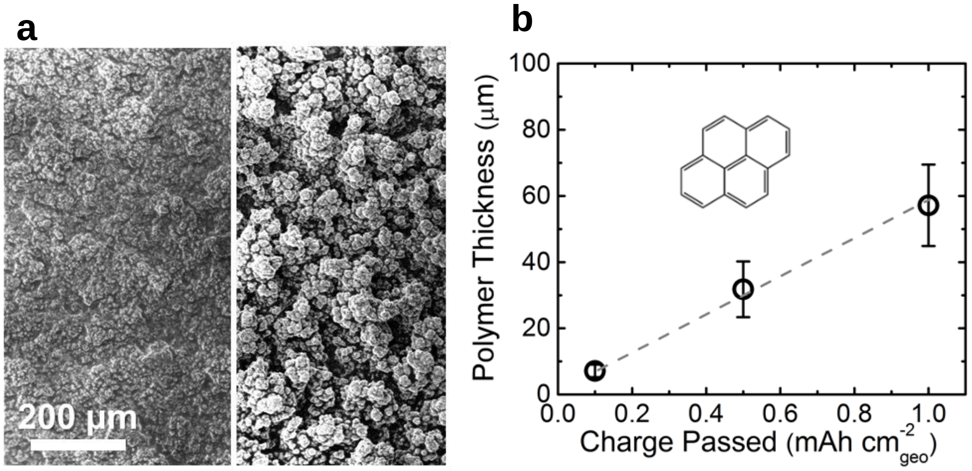

Figure 11. (a) Helium ion microscope images of polymerized pyrene on ITO coated glass. Sample was electropolymerized at 4.3 V versus Li/Li^+ for **(Left)** 0.1 mAh cm^{-2} resulting in a thickness and RMS Roughness of 7.1 and 5.1 µm, respectively and for **(Right)** 1 mAh cm^{-2} resulting in a thickness and RMS Roughness of 57.1 and 17.9 µm, respectively. **(b)** Thickness of polymerized pyrene on ITO coated glass slides as function of charge passed.

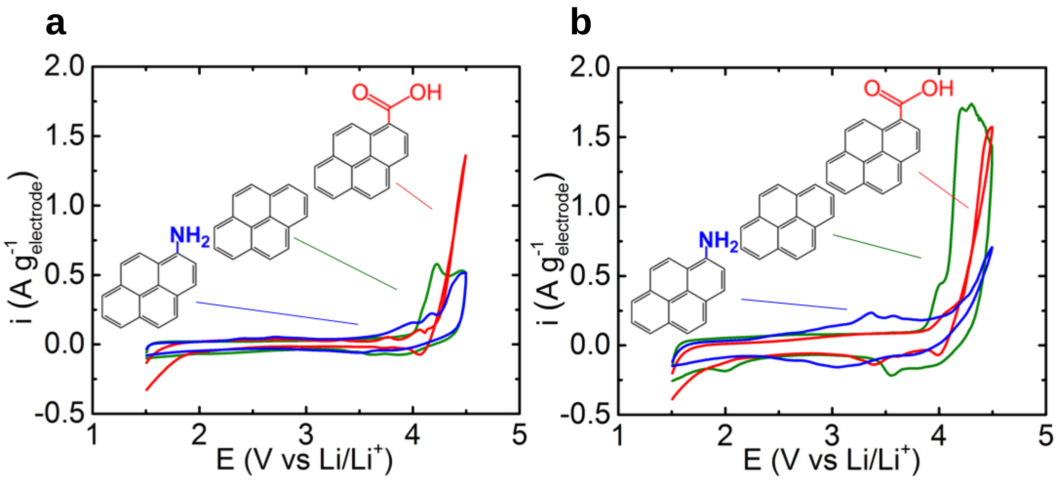

Figure 12. First cyclic voltammogram between 1.5 – 4.5 V versus Li/Li$^+$ at 1 mV s^{-1}, showing polymerization reaction of pyrene derivatives on **(a)** FWNT substrates and **(b)** oxidized-FWNT substrate in a two-electrode cell with lithium metal as the negative electrode, Celgard 2500 separators, and EC:DMC (3:7 volume ratio) with 1 M $LiPF_6$.

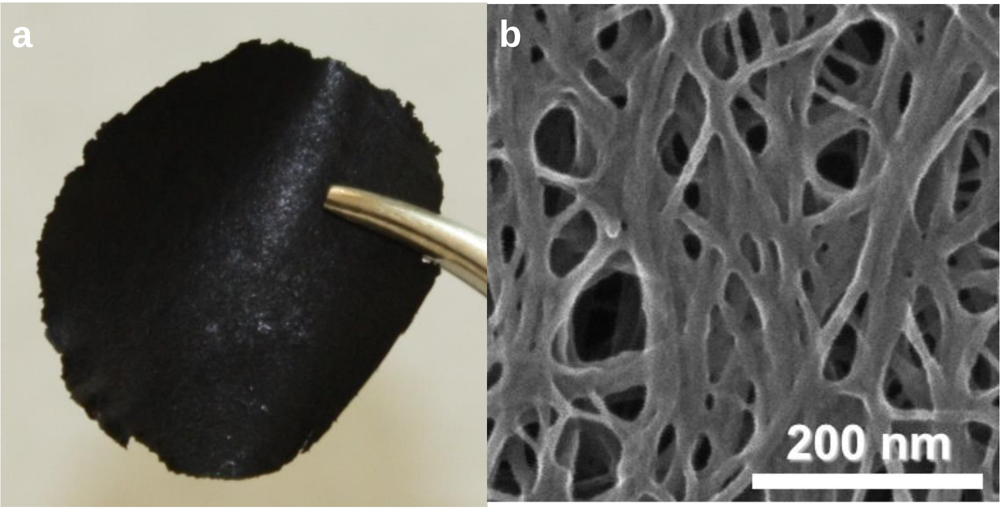

Figure 13. (a) Digital image of freestanding oxidized FWNT/aminopyrene electrode after electropolymerization through five cycles at 1 mV s^{-1} and five cycles at 5 mV s^{-1} between 1.5 – 4.5 V versus Li/Li^{+}. **(b)** Helium ion microscope image of oxidized FWNT/aminopyrene electrode after polymerization.

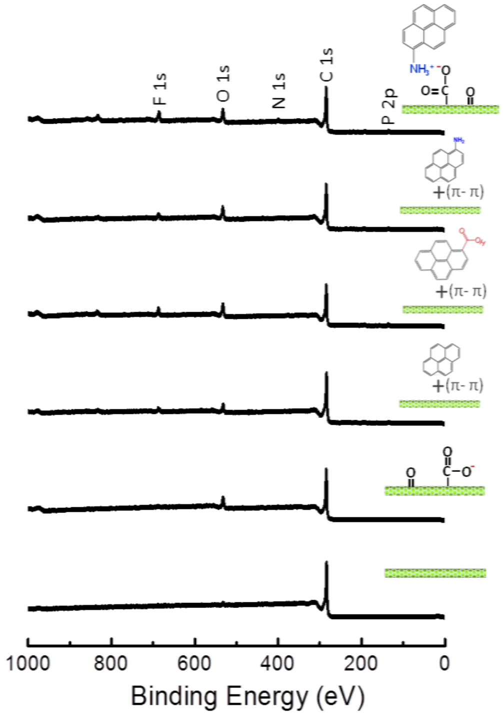

Figure 14. Survey X-ray photoelectron spectrum of (from bottom to top) pristine FWNT, oxidized FWNT, FWNT/pyrene, FWNT/pyrenecarboxylic acid, FWNT/aminopyrene, and oxidized FWNT/aminopyrene electrodes.

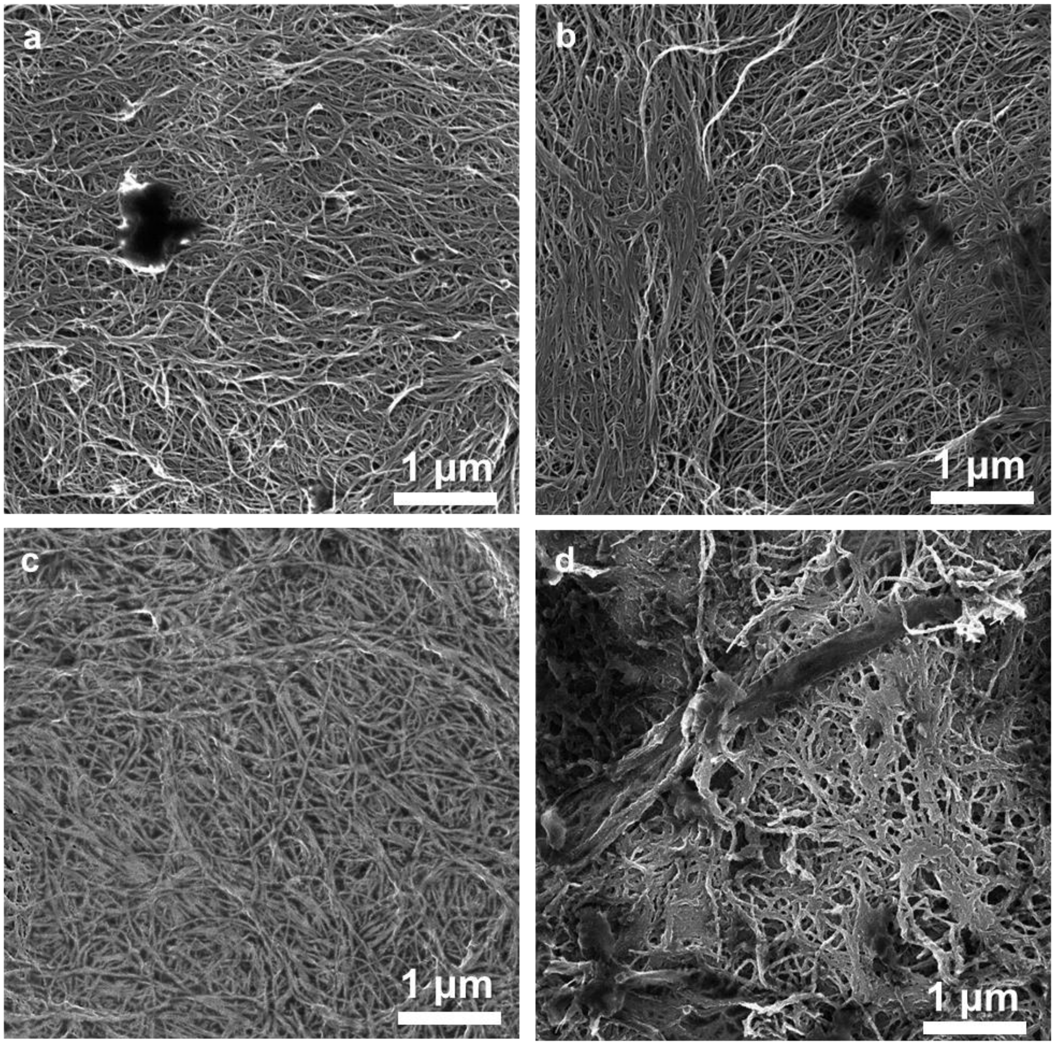

Figure 15. Helium-ion microscope images of **(a)** FWNT/polymerized pyrene, **(b)** FWNT/polymerized aminopyrene, **(c)** oxidized FWNT/polymerized pyrene, and **(d)** oxidized FWNT/polymerized pyrenecarboxylic acid electrodes. Pyrene and aminopyrene on the pristine FWNTs showed similar inhomogeneous spheres or irregular coatings on specific locations of the FWNT matrix. The polymerized pyrene and pyrenecarboxylic acid on the oxidized FWNTs showed a more uniform distribution of polymer compared to the pristine FWNT substrates, but they also included some irregular coatings compared to the polymerized aminopyrene on the oxidized FWNT.

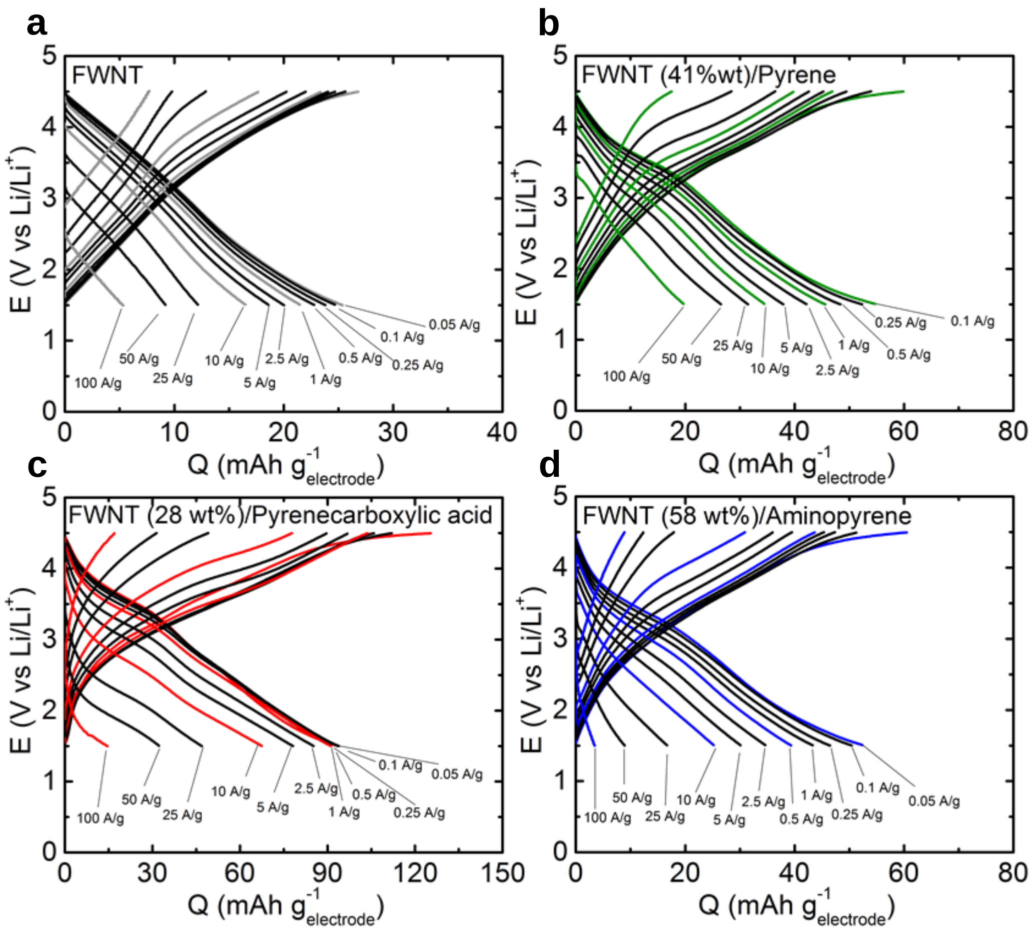

Figure 16. Galvanostatic rate capability of polymerized pyrene derivatives within FWNT substrate. **(a)** Pristine FWNT, **(b)** FWNT/pyrene (41 wt% FWNT) , **(c)** FWNT/pyrenecarboxylic acid (28 wt% FWNTs), and **(d)** FWNT/aminopyrene (58 wt% FWNT). The voltage window was 1.5–4.5 V versus Li/Li^+ and the current densities ranged from 0.05 A g^{-1} to 100 A g^{-1}. Prior to charge and discharge, the cells were held at a constant voltage of 1.5 and 4.5 V versus Li/Li^+, respectively, for 30 minutes.

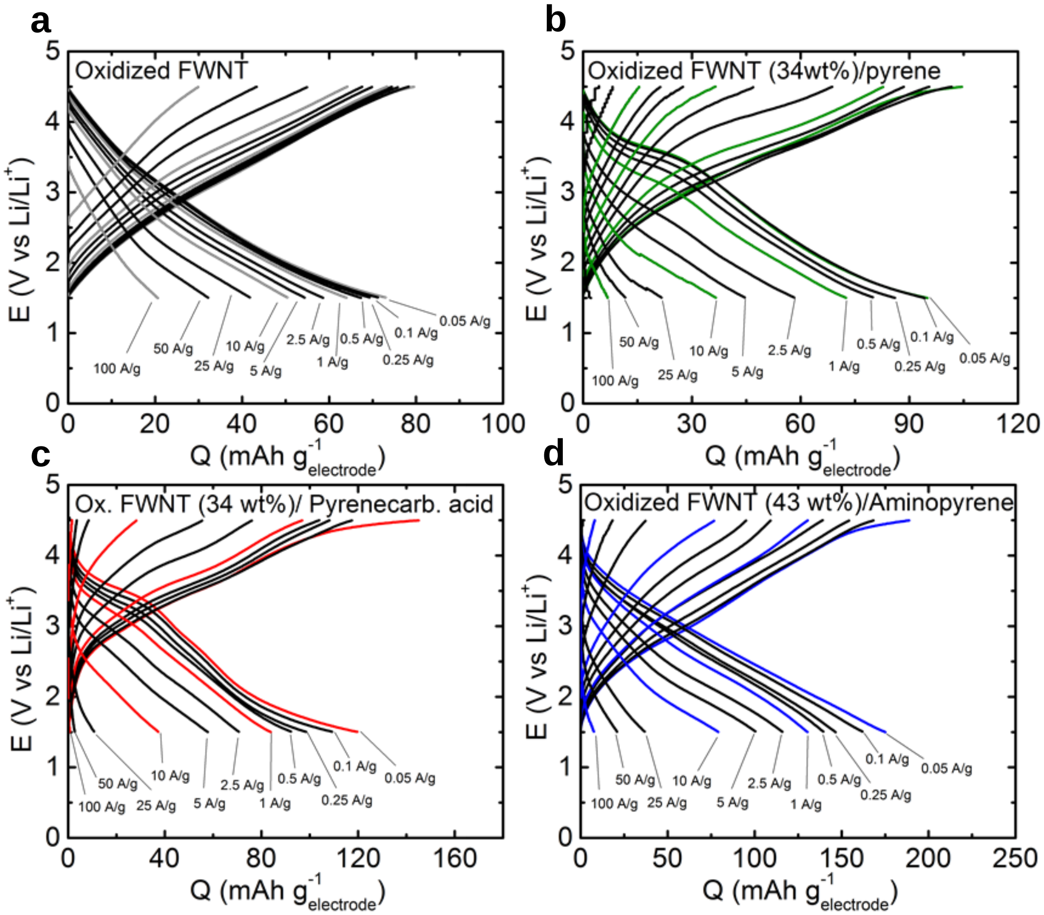

Figure 17. Galvanostatic rate capability of polymerized pyrene derivatives within **(a)** oxidized FWNTs, **(b)** oxidized FWNT/polypyrene (34 wt% FWNT), **(c)** oxidized FWNT/pyrenecarboxylic acid (34 wt% FWNT), and **(d)** oxidized FWNT/aminopyrene (43 wt% FWNT). The voltage window was 1.5–4.5 V versus Li/Li^+ and the current densities ranged from 0.05 A g^{-1} to 100 A g^{-1}. Prior to charge and discharge, the cells were held at a constant voltage of 1.5 and 4.5 V versus Li/Li^+, respectively, for 30 minutes.

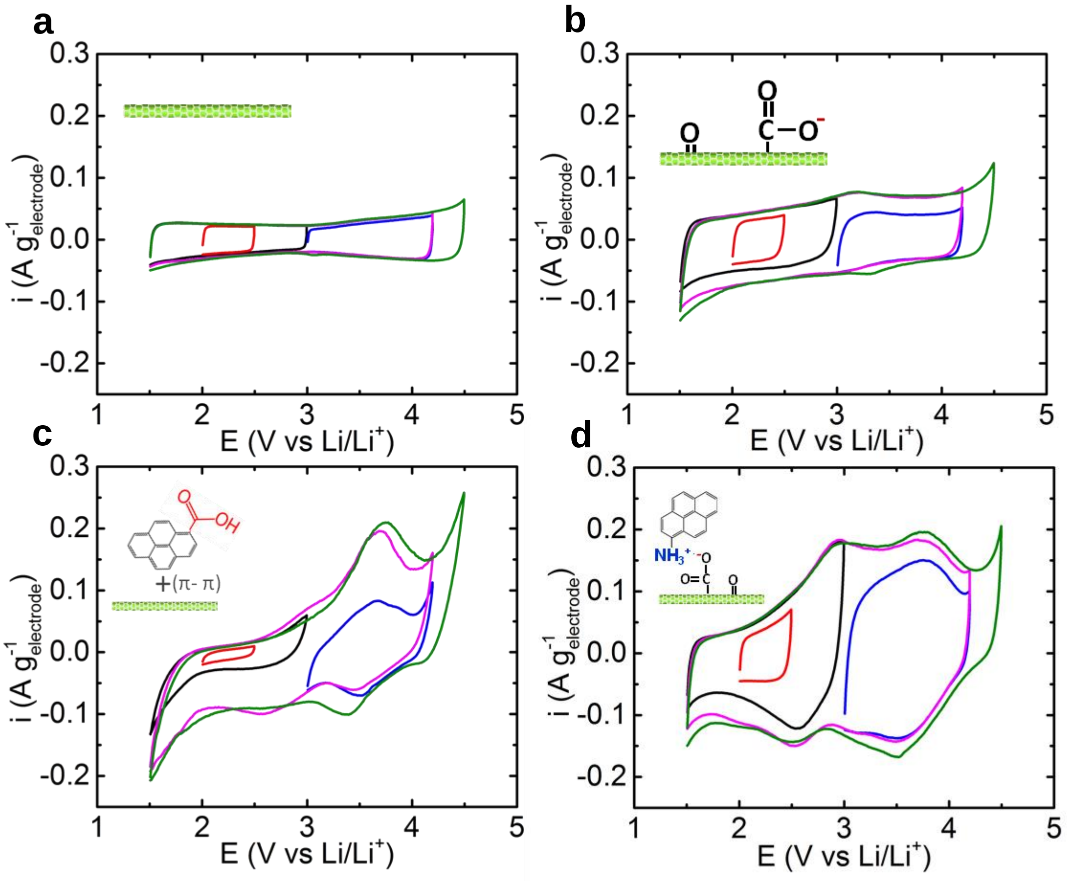

Figure 18. Potential-dependent cyclic voltammograms of **(a)** FWNT, **(b)** oxidized FWNT, **(c)** FWNT/pyrenecarboxylic acid, **(d)** oxidized FWNT/aminopyrene electrodes. The examined potential windows were 2.0 – 2.5 V versus Li/Li^+ (red), 1.5 – 3.0 V versus Li/Li^+ (black), 3.0 – 4.2 V versus Li/Li^+ (blue), 1.5 – 4.2 V versus Li/Li^+ (purple), and 1.5 – 4.5 V versus Li/Li^+ (green) at a scan rate of 1 mV s^{-1}.

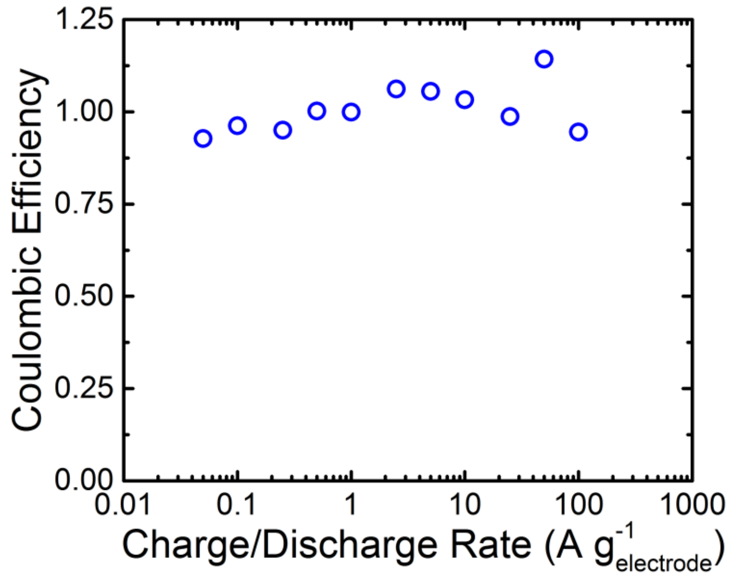

Figure 19. (a) Coulombic efficiency of oxidized FWNTs/aminopyrene (43 wt% FWNT) as a function of the charge/discharge rate.

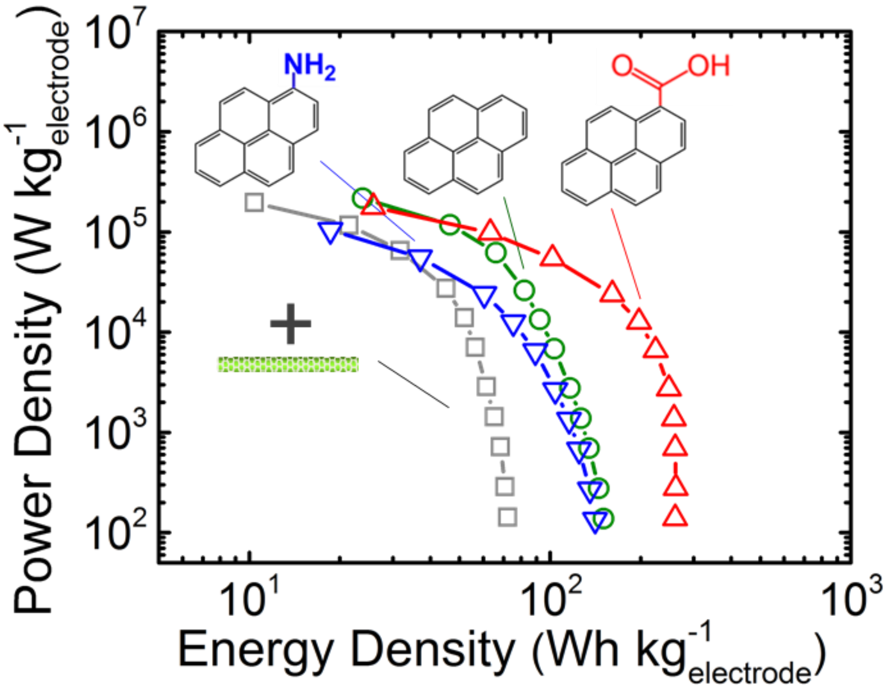

Figure 20. Ragone plot comparing gravimetric energy and power densities of polymerized pyrene derivatives on FWNT substrates with lithium negative electrodes. Shown above are pristine FWNT (gray), FWNT/pyrene (41 wt% FWNT) (green), FWNT/pyrenecarboxylic acid (28 wt% FWNT) (red), and FWNT/aminopyrene (58 wt% FWNT) (blue) electrodes. The densities of the electrodes range from 0.2 – 0.7 g cm^{-3}.

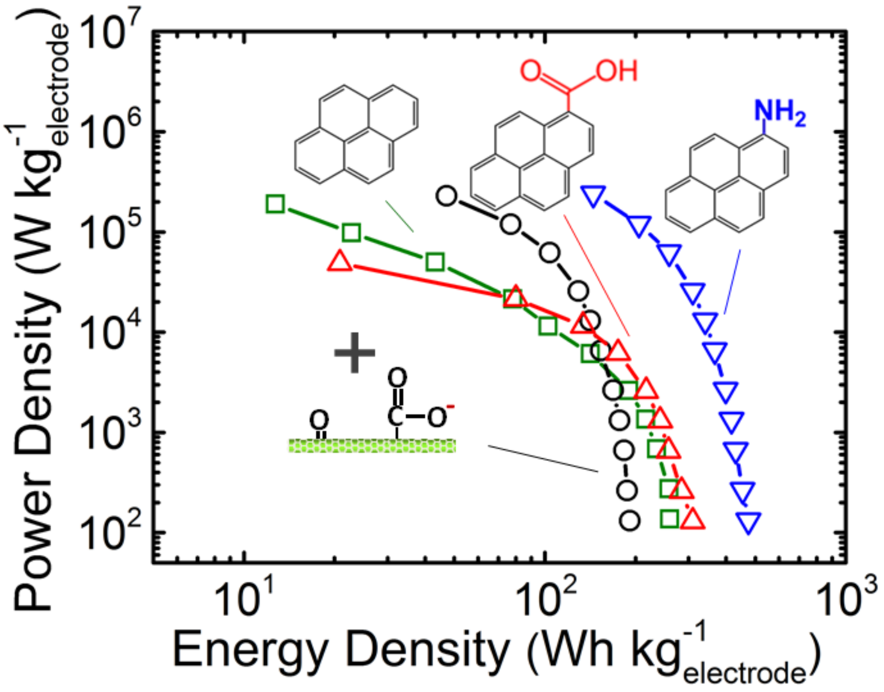

Figure 21. Ragone plot comparing gravimetric energy and power densities of polymerized pyrene derivatives on oxidized FWNT substrates with lithium negative electrodes. Shown above are oxidized FWNT (black), oxidized FWNT/polypyrene (34 wt% FWNT) (green), oxidized FWNT/pyrenecarboxylic acid (34 wt% FWNT) (red), and oxidized FWNT/aminopyrene (66 wt% FWNT) (blue) electrodes. The densities of the electrodes range from 0.4 – 0.7 g cm^{-3}.

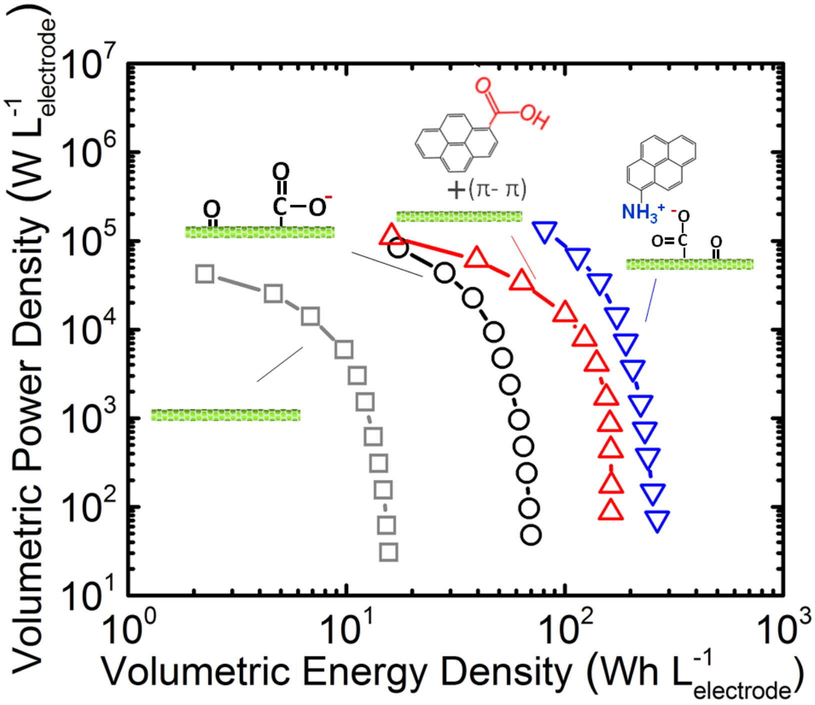

Figure 22. Volumetric energy and power densities of pristine FWNT (~0.2 g cm^{-3}) (gray), oxidized FWNT (~0.4 g cm^{-3}) (black), FWNT/pyrenecarboxylic acid (28 wt% FWNT, ~0.7 g cm^{-3}) (red), and oxidized FWNT/aminopyrene (66 wt% FWNT, ~0.6 g cm^{-3}) (blue) electrodes. The densities of the full electrodes are given above.

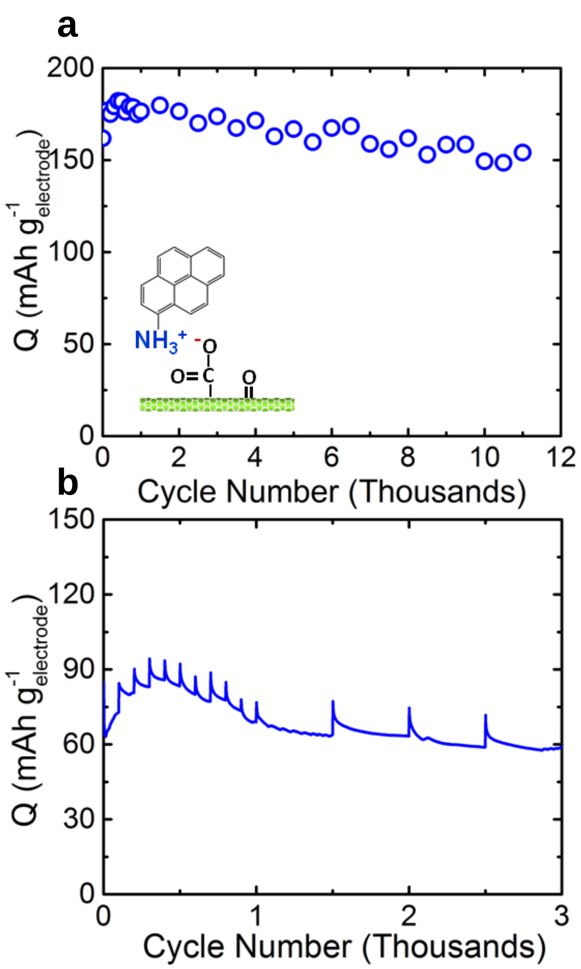

Figure 23. **(a)** Specific capacity of oxidized FWNTs/aminopyrene (43 wt% FWNT) electrode as a function of cycle number, which were measured at a current density of 0.1 A g^{-1} once every 100 cycles until 1000 total cycles and then once every 499 cycles. The voltage was held before slow-rate charge and discharge for 30 min. Within each 100 (for the first 1000 cycles) or 499 cycles (for the subsequent cycles), these cells were cycled under an accelerated rate of 10 A g^{-1}. We speculate the initial capacity increase comes from the electrolyte having additional time to diffuse into the pores of the electrode, allowing access to additional active material. Additionally, the capacity increase can be attributed to the continued polymerization of monomers remaining on the surface of the electrode and in the electrolyte. **(b)** The specific capacity measured during the accelerated cycles at 10 A g^{-1}. One can see that there is a similar decrease in capacity at high rates as there is at low rates. Additionally, the high-rate-specific capacities show hysteresis from the slow discharge cycles.

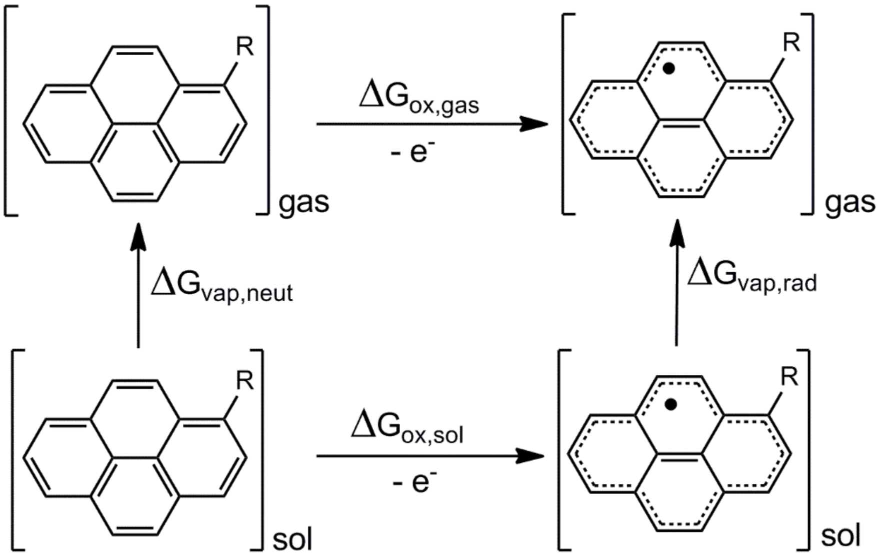

Figure 24. Thermodynamic cycle used for calculating relative HOMO energies and redox potentials.

Supplementary Tables

Electrode	Elemental Composition (Atomic %)					
	Carbon	Oxygen	Fluorine	Phosphorus	Nitrogen	Lithium
FWNT	98%	2%	0%	0%	0%	0%
Oxidized FWNT	89%	9%	1%	<1%	1%	0%
FWNT/Pyrene	91%	7%	2%	1%	0%	0%
FWNT/Pyrenecarboxylic Acid	81%	13%	5%	1%	0%	0%
FWNT/Aminopyrene	86%	10%	3%	<1%	1%	0%
Oxidized FWNT/Aminopyrene	76%	11%	6%	1%	2%	4%

Table 1. Elemental composition of selected electrodes measured through X-ray photoelectron spectroscopy. As expected, carbon 1s peaks were found in all cases from both the polymer and carbon nanotubes. The oxidized FWNTs had additional oxygen content as expected from the pristine FWNTs. The samples with polymerized pyrene derivatives contained carbon, oxygen, nitrogen, fluorine, and phosphorus peaks. The fluorine and phosphorus peaks result from inserted anions into the polymers or residual salt in the electrolyte (these sample were rinsed in dimethyl carbonate, but the samples are porous).

Supplementary Methods

Electrochemical measurements and calculations

All experiments on glassy carbon (GCE, CH Instrument Inc) and indium tin oxide working electrodes (Rs = 4–8Ω, Delta Technologies, LTD) were carried out in a three-electrode cell at room temperature using a Biologic (SP300 or VSP300) potentiostat, lithium as the counter electrode, and lithium as the reference electrode. The working electrodes were polished with Buehler 5 µm alumina polishing suspension, rinsed with distilled water and ethanol, and dried prior to use. The only exception was for the electrochemical quartz crystal microbalance measurements where a Au coated quartz crystal electrode and cell from Biologic was used in conjunction with a Seiko quartz crystal analyzer and a lithium counter electrode. Naphthalene (99.7%, Fluka), phenanthrene (99.5%, Aldrich), pyrene (99%, Aldrich), and perylene (99.5%, Aldrich) were dispersed in the stated concentrations within EC:DMC (3:7 volume ratio) with 1 M $LiPF_6$ or 1 M $LiBF_4$ (<20 ppm H_2O, BASF) or was used within EC:DMC (3:7 volume ratio) with dried 1 M $LiClO_4$ (99.99%, Aldrich).

All experiments on FWNT electrodes were done in two-electrode cells (Tomcell, Japan) assembled inside an argon-filled glovebox (Vacuum Atmosphere Co., O_2 level below 1 ppm and H_2O level below 1 ppm). In all cases, as assembled cells were allowed to rest for at least 8 hours prior to testing in order to allow full wetting of porous films by the electrolyte. The cells consisted of a free-standing vacuum-filtered FWNT positive electrode, a lithium metal negative electrode, two porous Celgard 2500 separators (25 µm thickness and 64 nm average pore diameter), and 1 M $LiPF_6$ in an EC:DMC (3:7 volume ratio) electrolyte. The electrodes were tested electrochemically using a Solartron 1470 test unit in the voltage range 1.5 – 4.5 V versus Li/Li^+. For galvanostatic rate capability tests, the current density ranged from 0.05 to 100 A g^{-1}, corresponding to the C-rates from 0.29 C to 580 C in the case of the oxidized FWNT/aminopyrene electrode. The voltage was held constant at either 1.5 or 4.5 V versus Li/Li^+ for 30 minutes prior to charge or discharge, respectively. Galvanostatic capacities can vary depending on the amount and morphology of polymer coating. For example, the case with the largest variability—for the oxidized FWNT/aminopyrene electrode—the capacity can range from ~100-180 mAh $g^{-1}_{electrode}$ depending on the amount of polymer coating. The loading of the polymer

coating has been found to vary from ~21-63% of total weight of the electrode. Cycling tests consisted of galvanostatic cycling at 10 A g^{-1} for 99 cycles, followed by a slower charge and discharge cycle at 0.1 A g^{-1} every 100 cycles up to 1000 cycles, with a 30 min voltage hold at 1.5 V or 4.5 V versus Li/Li^{+} prior to low-rate charge or discharge, respectively. For cycle numbers between 1000 and 11,000, 499 cycles were performed for every slower charge and discharge cycle.

All performance metrics were normalized to the total weight of the positive electrode, including both the FWNT and polymer coating. Energy densities are calculated based on the integration of the discharge profiles of the potential as a function of specific capacity:

$$e = \int V dq \qquad (1)$$

Where e is the specific energy, V is the voltage of the cell, and q is the specific discharge charge. The average power density during discharge is reported on all Ragone plots and is calculated as the ratio of the energy density to the total time for discharge. The average specific capacitance is determined from the discharge profile as:

$$\bar{c} = \Delta q/\Delta V \qquad (2)$$

Where $\bar{c}$ is the average specific capacitance, Δq is the specifc discharge capacity (note one must multiply a value in mAh g^{-1} by 3.6 to convert to C g^{-1} for calculations of F g^{-1}), and ΔV is the difference in potential from the start to the end of discharge (3 V in the case of this research).

Sample preparation

Sub-millimeter long few-walled nanotubes (FWNTs) (6–10 nm diameter, 0.4 mm length, 99 wt% purity, 400 m^2/g specific surface area, triple walled on average) were synthesized by CVD in a single fluidized bed reactor.[1]

Functionalization of FWNT was performed using a previously reported method.[2] Briefly, functionalized FWNTs were prepared by oxidizing pristine FWNTs in a mixture of H_2SO_4 (96.5%, Aldrich) and HNO_3 (70%, Aldrich) solution (3:1 volume ratio) at 70 °C for 2 h. The functionalized FWNTs were washed in a ~5% by volume HCl solution and dried in air.

FWNTs or oxidized FWNTs were mixed with stated molecules (pyrene (99%, Aldrich), 1-aminopyrene (97%, Aldrich), 1-pyrenecarboxylic acid (97%, Aldrich)) in various ratios, dispersed in Milli-Q deionized water and ethanol at a concentration of 0.2 mg mL^{-1}. Binder-free electrodes were synthesized through vacuum filtration on Celgard 2500 membranes. Electrodes were further dried at 70 °C under vacuum and polymerized in a two-electrode cell typically through 5 CV cycles at 1 mV s^{-1} and subsequent 5 CV cycles at 5 mV s^{-1} between 1.5 - 4.5 V versus Li/Li^{+}.

Characterization

The microstructure of the polymer electrodes were investigated using a scanning helium-ion microscope (Orion Plus Helium-Ion Microscope from Carl Zeiss) operating at 30 kVs and a JEOL 2010 transmission electron microscope (TEM) with a LaB6 thermal emission electron gun was used for high-resolution imaging. The microscope was operated at 200 kV and was able to ultimately achieve 0.19 nm point-to-point resolution. Raman measurements were taken with a Horiba Scientific Labram HR unit. FTIR measurements were taken on a Bruker Vertex 70.

For identifying the surface chemistry of the polymer electrodes, a Physical Electronics Versaprobe II X-ray Photoelectron Spectrometer was used. The relative sensitivity factors used to scale the peaks of C 1s, N 1S, O 1s, F 1s, and P 2p were 58.791, 93.486, 137.408, 187.576, and 113.106, respectively.

The thickness of the polymer coated on the indium tin oxide substrate was determined by scratching away the polymer to the substrate and averaging the thickness at four locations across the step change in height using a Tencor P-16 Surface profilometer. The densities of the electrodes were determined by measuring the mass and volume of the polymer and FWNT substrate. The volume of each FWNT electrode was determined by multiplying the thickness and the geometric area of the electrode.

Computation

All calculations were carried out with the ORCA program package. Every unrestricted Kohn-Sham DFT calculation employed the Perdew-Burke-Ernzerhof (PBE) generalized gradient approximation (GGA) functional in conjunction with the def2-TZVP basis set on every atom. The RI approximation was used (RIJONX) with the def2-TZVP/J auxiliary

basis set. The calculations proceeded with "verytightscf" and "veryslowconv" convergence criteria. The EC:DMC (3:7 volume ratio) solution was simulated using the COSMO solvation model. The refractive index (δ) of 35.4 and dielectric constant (ε) of 1.38 were calculated from the known values,[3-6] assuming the contribution of each solvent to the δ and ε of the mixture was proportional to their molar fraction. Numerical frequency calculations confirmed the minima contained only real frequencies. The rotational energy correction was manually subtracted using a symmetry number of 1 for all substituted pyrenes and a symmetry number of 2 for pyrene, naphthalene, phenanthrene, and perylene.

For the neutral and oxidized form of each pyrene derivative, geometries were optimized in both the solution and gas phases. The output energies were plugged into the thermodynamic cycle (Equation 3 and Supplementary Fig. 24), which is commonly used in the DFT calculations of reduction potentials.[7] The energies were converted from hartrees to eV at T = 295 K. Due to our method of allowing the relaxed geometries to optimize in all phases and not just the gas phase, calculating the gas phase energies proved unnecessary, as the energy difference between the species in solution was identical to the energy difference calculated from the thermodynamic cycle in all cases. Therefore, in the cases of perylene, pyrene, phenanthrene, and napthalene only the solution optimized structures and energies were calculated to determine the free energy of oxidation.

$$\Delta G_{ox,sol} = \Delta G_{vap,neut} + \Delta G_{ox,gas} - \Delta G_{vap,rad} \qquad (3)$$

The difference in energy between the neutral and oxidized species in solution was converted into a potential, all potentials and energy differences were referenced to those of pyrene.

Supplementary References

1. Kim DY, *et al.* Sub-millimeter-long carbon nanotubes repeatedly grown on and separated from ceramic beads in a single fluidized bed reactor. *Carbon* **49**, 1972-1979 (2011).

2. Lee SW, *et al.* Self-standing positive electrodes of oxidized few-walled carbon nanotubes for light-weight and high-power lithium batteries. *Energy Environ. Sci.* **5**, 5437-5444 (2012).

3. Seward RP, Vieira EC. The Dielectric Constants of Ethylene Carbonate and of Solutions of Ethylene Carbonate in Water, Methanol, Benzene and Propylene Carbonate. *J. Phys. Chem.* **62**, 127-128 (1958).

4. Tundo P, Selva M. The Chemistry of Dimethyl Carbonate. *Acc. Chem. Res.* **35**, 706-716 (2002).

5. NIST/TRC Web Thermo Tables. 2012 Available from: http://wtt-pro.nist.gov.

6. Rodríguez A, Canosa J, Tojo J. Physical Properties of the Ternary Mixture Dimethyl Carbonate + Methanol + Benzene and Its Corresponding Binaries at 298.15 K. *J Chem. Eng. Data* **44**, 1298-1303 (1999).

7. Konezny SJ, *et al.* Reduction of Systematic Uncertainty in DFT Redox Potentials of Transition-Metal Complexes. *J. Phys. Chem. C* **116**, 6349-6356 (2012).

8. Lu G, Shi G. Electrochemical polymerization of pyrene in the electrolyte of boron trifluoride diethyl etherate containing trifluoroacetic acid and polyethylene glycol oligomer. *J. Electroanalyt. Chem.* **586**, 154-160 (2006).
